# Far-red chlorophyll d clusters extend photosystem I absorption toward the red limit

**DOI:** 10.1126/sciadv.aed7355

**Published:** 2026-06-10

**Authors:** Thomas J. Oliver, Eduard Elias, Giovanni Consoli, Ho Fong Leong, Violeta Cordón-Preciado, Andrea Fantuzzi, Tanai Cardona, A. William Rutherford, Roberta Croce

**Affiliations:** ^1^ Department of Physics and Astronomy, Vrije Universiteit Amsterdam, Amsterdam, Netherlands.; ^2^ Department of Life Sciences, Imperial College London, London, UK.; ^3^ School of Biological and Behavioural Sciences, Queen Mary University of London, London, UK.

## Abstract

Oxygenic photosynthesis is usually limited to visible light, but the marine cyanobacterium *Acaryochloris marina* pushes this boundary by harvesting far-red photons with chlorophyll d. The best-studied strain, MBIC11017, unexpectedly lacks low-energy chlorophylls (“red forms”) in photosystem I, limiting absorption beyond 740 nanometers. Here, we show that another strain, *A. marina* NIES-2412, has evolved a strategy to absorb far-red photons up to 760 nanometers. Combining time-resolved fluorescence spectroscopy with cryo–electron microscopy at 2.64-angstrom resolution, we identify two distinct classes of chlorophyll d red forms in its photosystem I. One class originates from classical charge-transfer–exciton mixing, while the other arises purely from excitonic interactions. Mapping all 96 chlorophylls d reveals the precise pigments responsible for these far-red states. We also uncover a previously unreported subunit, PsaX2, which stabilizes the photosystem I complex and shapes pigment geometry and energetics to enable the formation of red forms. Last, we show that the protein modifications responsible for binding and tuning these red forms are widespread across the *Acaryochloris* genus but not within the model MBIC11017 strain. Far-red photons lie close to the energetic limit of oxygenic photosynthesis; their efficient use therefore requires fine-tuning of the photosynthetic machinery. To our knowledge, our findings provide the structural and mechanistic basis of one of the most red-shifted photosystem I complexes identified to date, highlighting a distinct adaptive strategy in far-red light environments and offering design principles for extending photosynthesis in crops into the infrared.

## Teaser

The cyanobacterium *A. marina* shows how photosynthetically active radiation can be expanded into the far-red spectral region.

## INTRODUCTION

While most cyanobacteria perform oxygenic photosynthesis using chlorophyll a (Chl a), *Acaryochloris marina* uses the red-shifted Chl d. Chl d differs from Chl a by the presence of a C3 formyl group rather than a vinyl group, resulting in a ~30-nm red shift. This enables *A. marina* to thrive in shaded environments enriched in far-red light (FRL, 700 to 800 nm). Although the enzyme responsible for Chl d biosynthesis is unknown, the impact of its incorporation into photosynthetic complexes has been the subject of a number of studies, primarily using the type strain *A. marina* MBIC11017 (*1*–*10*) (hereafter MBIC11017).

Photosystem I (PSI) is one of the multisubunit pigment-protein complexes that make up the photosynthetic machinery of oxygenic phototrophs. It is formed by the 2 pseudosymmetric core subunits, PsaA and PsaB, alongside 9 to 10 additional subunits, binding ~95 Chls. Most of these Chls act as antenna pigments, funneling excitation energy to the reaction center (RC), where charge separation occurs. Despite using Chl d, with a lower excited-state energy (*11*), MBIC11017 PSI maintains a quantum efficiency of charge separation of ~98% (*12*), as high as in Chl a–containing PSI (*13*). However, unlike most Chl a–PSI, MBIC11017 PSI does not contain any “red forms,” i.e., Chl multimers absorbing at energies lower than the primary donor of the complex, in this case P_740_. Red forms arise from the strong excitonic interaction between two or more Chls, as well as contribution from charge-transfer (CT) states (*14*–*16*). They are common in PSI from all organisms (*17*, *18*), extending their absorption into the far red (*19*, *20*), which is beneficial in shaded environments or dense cultures. Because of their low energy, red forms act as local traps, necessitating uphill excitation energy transfer (EET) to the RC for charge separation to occur. The number and site energy of red forms within PSI roughly correlate with its overall trapping time (*21*).

The absence of red forms in MBIC11017 PSI minimizes its overall trapping time and increases its quantum efficiency of charge separation. This likely reflects adaptation to a spectral niche more enriched in white light than FRL (*12*, *22*). It has recently emerged that there exists a large diversity of *Acaryochloris* strains, many of which exhibit substantially more red-shifted absorption and emission spectra, both in vivo and in purified PSI (*23*, *24*). The proteins and Chls responsible for extending their PSI absorption further in the far-red remain unknown. By incorporating low-energy Chl forms into PSI, which absorb near the energetic limit of oxygenic photosynthesis, these strains risk lowered excitation trapping efficiencies and reduced photosynthetic performance.

Here, we have investigated PSI from *A. marina* NIES-2412 (hereafter referred to as NIES-2412), a strain that displays far-red spectral properties distinct from MBIC11017. By studying this strain, we aim to clarify how PSI can incorporate low-energy chlorophyll forms, which structural features enable their stabilization, and how they influence the performance of the complex. This is essential information for designing an efficient strategy to increase far-red absorption in crops.

## RESULTS

### Spectroscopy of NIES-2412 PSI

**Fig. 1.**
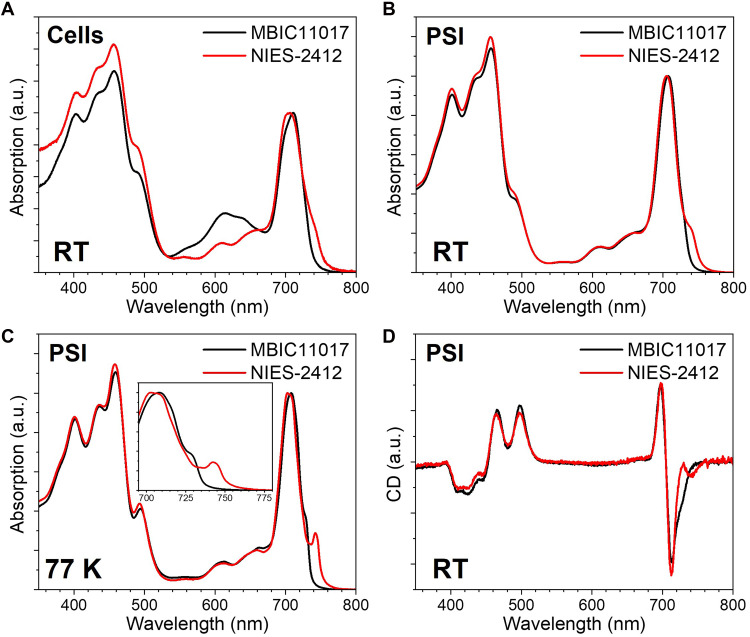
Absorption and circular dichroism spectra of *A. marina* NIES-2412 cells and the isolated PSI complex compared to *A. marina* MBIC11017. (**A**) Room temperature (RT) absorption spectra of NIES-2412 and MBIC11017 cells. Spectra were normalized to their maxima at ~706 to 710 nm. (**B**) RT absorption spectra of NIES-2412 and MBIC11017 isolated PSI complexes. Spectra are normalized to their maxima at ~708 nm. (**C**) Absorption spectra (77 K) of NIES-2412 and MBIC11017 isolated PSI complexes. Spectra are normalized to their maxima at ~703 to 708 nm. Inset shows the *Q*_Y_ region of the spectrum between 705 and 775 nm. (**D**) Circular dichroism (CD) spectra of NIES-2412 and MBIC11017 isolated PSI complexes. The absence of the ~728-nm shoulder in NIES-2412 is largely due to the compensation of a new positive band coupled with the 742-nm feature. Spectra are normalized to their integrated absorption spectrum between 670 and 800 nm. a.u., arbitrary units.

Absorption spectra of NIES-2412 and MBIC11017 cells differ markedly (Fig. 1A). NIES-2412 cells show a shoulder to the main *Q*_Y_ peak, extending absorption to ~760 nm, a feature absent in MBIC11017. Its *Q*_Y_ absorption maximum (~706 nm) is slightly blue-shifted compared with MBIC11017 (~710 nm). NIES-2412 cells also lack absorption between 550 and 650 nm, indicating the absence of phycobilisomes, unlike MBIC11017.

To investigate the origin of the red-shifted shoulder, we isolated PSI from NIES-2412 (fig. S1). The room temperature (RT) absorption spectrum of NIES-2412 PSI (Fig. 1B) still exhibits the red shoulder, which at 77 K narrows (Fig. 1C), appearing as a distinct band at 743 nm. Its *Q*_Y_ absorption maximum is blue-shifted relative to MBIC11017 PSI (~703 nm versus ~708 nm) and lacks the small shoulder at 728 nm. The absorption spectra of pigments extracted from each PSI complex are essentially identical (fig. S2A), with maxima at ~693 nm indicative of Chl d. Fitting of these spectra with the isolated Chl a and Chl d spectra shows that NIES-2412 PSI contains only Chl d (fig. S2B), while MBIC11017 has a small quantity of Chl a (2.6%) (fig. S2C).

Gaussian deconvolution of the NIES-2412 PSI 77 K absorption spectrum (fig. S3) shows that the red-shifted absorption peak can be described by three bands: a narrow band centered at 744 nm [full width at half maximum (FWHM) = ~8.5 nm], a broad one at 745 nm (FWHM = 22 nm), and a much broader one at 750 nm (FWHM = 47 nm). Considering that the NIES-2412 PSI complex contains 96 chlorins per monomer (see “Structure of NIES-2412 PSI” section below), the area under these bands corresponds to 3.6, 3.6, and 1.5 Chl d molecules. However, the P_740_ absorption, with an oscillator strength of ~2 Chls d (*6*), is also described by the 744- or 745-nm Gaussians.

While the circular dichroism (CD) spectra of NIES-2412 PSI and MBIC11017 PSI (Fig. 1D) are very similar in the Soret region, they differ in the *Q*_Y_ region. The NIES-2412 PSI spectrum has a distinct negative band at ~742 nm, absent in MBIC11017, which is indicative of an excitonic interaction that creates a low-energy red form.

**Fig. 2.**
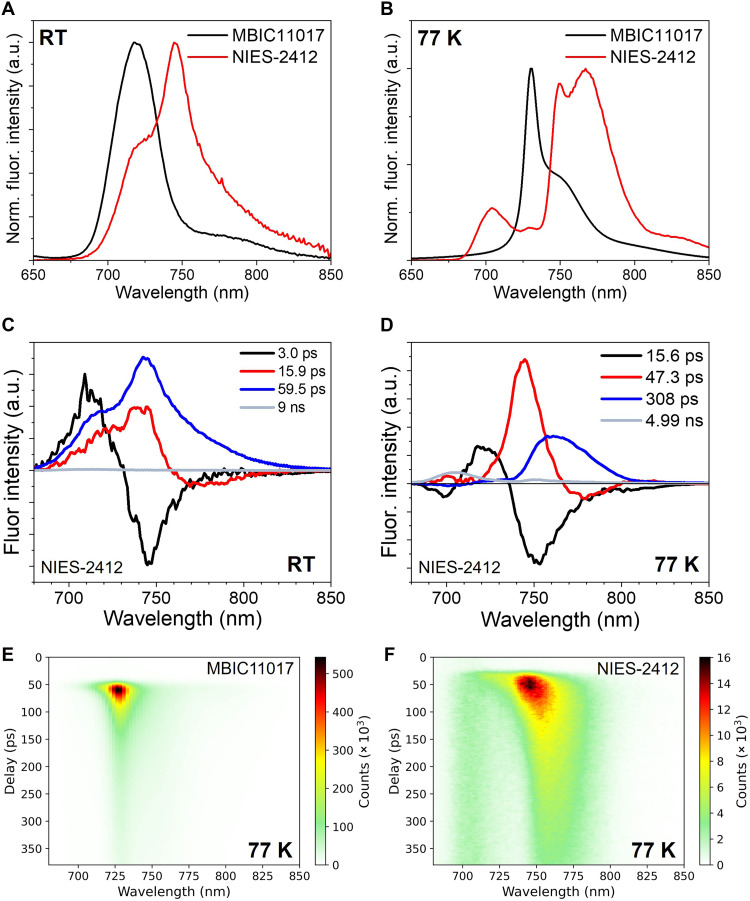
Steady-state and time-resolved fluorescence spectra from *A. marina* NIES-2412 PSI. (**A**) Room temperature (RT) fluorescence emission spectra of NIES-2412 and MBIC11017 [taken from Oliver *et al.* (*12*)] isolated PSI complexes. Spectra were normalized to their emission maxima. (**B**) Fluorescence emission spectra (77 K) of NIES-2412 and MBIC11017 [taken from Oliver *et al.* (*12*)] isolated PSI complexes. Spectra were normalized to their emission maxima. (**C**) Decay-associated spectra from global analysis of RT time-resolved fluorescence data from the NIES-2412 isolated PSI complex. (**D**) Decay-associated spectra from global analysis of 77-K time-resolved fluorescence data from the NIES-2412 isolated PSI complex. (**E**) Two-dimensional color map of time-resolved fluorescence (TRF) data of MBIC11017 PSI at 77 K, excited at 400 nm, and detected in the chlorophyll *Q*_Y_ region (λ > 650 nm) with a streak camera setup. (**F**) Two-dimensional color map of TRF data of NIES-2412 PSI at 77 K. a.u., arbitrary units.

The emission spectrum of NIES-2412 PSI is red shifted relative to MBIC11017 PSI (Fig. 2A). At RT, NIES-2412 PSI emits at 720 and 744 nm with a broad tail beyond 800 nm, compared to a 720-nm maximum for MBIC11017 (Fig. 2A). At 77 K, the maximum shifted to 767 nm, 36 nm more red shifted than MBIC11017 (Fig. 2B). Additional bands at ~700 and 750 nm likely originate from free Chl d and disconnected antennae that are overrepresented in the steady-state emission (see time-resolved fluorescence results).

To understand the effect of the red-shifted chlorophylls on excitation-energy trapping in NIES-2412 PSI, we measured its fluorescence decay using a streak camera setup. Global analysis of the data (Fig. 2C) shows two fast components corresponding to EET processes. The first (3 ps) shows EET to a pigment pool emitting at ~745 nm, while the second (~16 ps) shows EET to pigments emitting at ~770 nm. This component has a positive integrated area, indicating that energy trapping also occurs with a similar lifetime. The main energy trapping occurs in ~60 ps, almost twice as long as in MBIC11017 PSI (*12*). A minor nanosecond component arises from disconnected Chl d.

At 77 K, global analysis of time-resolved fluorescence (TRF) data also shows four components (Fig. 2D). The first two (~15 and ~50 ps) represent the same two EET processes observed at RT, which are slowed by effect of the temperature. Trapping from the 750-nm pool is observed in 50 ps. The third component (~300 ps) peaks at ~765 nm, indicating trapping from a low-energy form. The impact of this low-energy form on 77-K trapping dynamics is evident in the streak camera images (Fig. 2, E and F), which show a considerable increase in lifetime of NIES-2412 PSI compared with MBIC11017. A minor ~5-ns component arises from disconnected Chl d and antenna proteins.

**Fig. 3.**
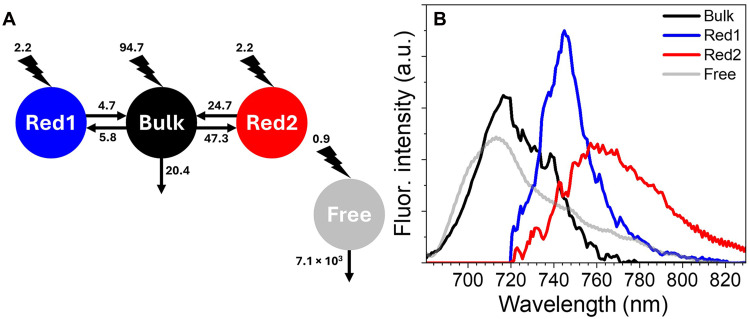
Target analysis results for the NIES-2412 PSI RT time-resolved fluorescence data. (**A**) Target kinetic scheme. The numbers above the lightning bolts indicate the fitted portion of initial excitation for the different compartments. The numbers connected to the arrows indicate the reciprocal of the fitted kinetic rates in picoseconds. (**B**) Species-associated spectra (SAS) for the fitted kinetic scheme. The Free compartment SAS has been smoothed for clarity. a.u., arbitrary units.

Typically, excitation energy dynamics within PSI complexes are described by a kinetic scheme in which excitation energy is trapped from a large number of bulk Chls, which are in thermal equilibrium with one or more red forms (*18*, *21*, *25*). Stepanov analysis (*26*, *27*), showing a close match between measured and calculated emission spectra (fig. S4), confirms this description for NIES-2412 PSI. The target analysis (Fig. 3, A and B) disentangled two red forms: Red1, peaking at 745 nm, and Red2, at 757 nm. The Bulk compartment, which has a spectrum very similar to the RT emission spectrum of MBIC1107 PSI (fig. S5), traps with a characteristic time constant of 20.4 ps.

Red1 is populated quickly from the Bulk (5.8 ps), whereas Red2 is populated on a slower timescale (47.3 ps). Because fewer Chls contribute to Red1 and Red2 than to the Bulk, their equilibria are shifted toward the Bulk, despite its higher energy. Considering these equilibria, the spectra of the compartments, and the total number of Chls per PSI, the number of Chls in each compartment is estimated to be ~5 for Red1 and ~1 for Red2 (Materials and Methods and table S1). These values are consistent with the Gaussian deconvolution (6.7 Chls d absorbing above 740 nm, excluding P_740_). The spectrum of Red2 is markedly broader than that of Red1, despite the fewer Chls that contribute to it, indicative of extensive CT character.

### Structure of NIES-2412 PSI

To investigate the molecular origin of the Red1 and Red2 pools, the NIES-2412 PSI structure was solved through cryo–electron microscopy (cryo-EM) single-particle analysis, yielding a map of trimeric PSI at a gold-standard Fourier shell correlation (GS-FSC) resolution of 2.64 Å (fig. S6 and table S2).

**Fig. 4.**
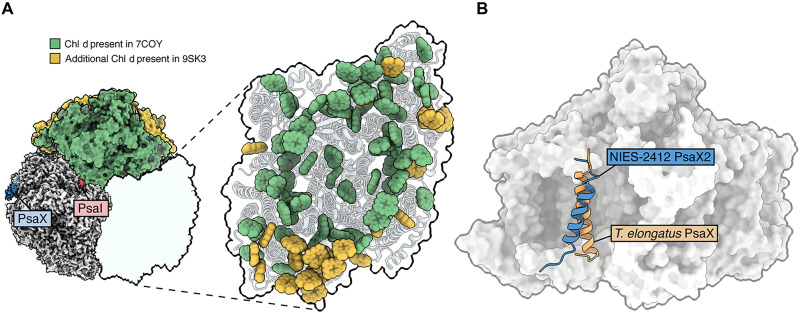
The structure of the trimeric PSI complex from *A. marina* NIES-2412, showing the location of all resolved Chl d, in comparison with *A. marina* MBIC11017 PSI. (**A**) View of the trimeric NIES-2412 ESP map from the cytoplasmic side. The map is shaded in yellow to show the additional areas that were resolved in NIES-2412 PSI, relative to MBIC11017 PSI [Protein Data Bank (PDB) ID: 7COY]. Green shading shows common areas, which were resolved in both structures. The ESP map of the NIES-2412 PSI monomer is also shown, with PsaX and PsaI labeled. The zoomed-in monomer shows the locations of the Chls within the NIES-2412 PSI monomer, with those shaded in green present in both the NIES-2412 and MBIC11017 structures and those shaded in yellow only present in NIES-2412. (**B**) Surface view from the plane of the membrane, looking at PsaB, showing the position of PsaX2. PsaX2 is colored blue, with *T. elongatus* PsaX overlaid in orange to show their relative positions.

The map reveals electrostatic potential (ESP) for 12 subunits coordinating 93 Chls d, 1 Chl d’, 2 pheophytins *a* (fig. S7), 21 α-carotenes, 2 phylloquinones, 3 Fe_4_S_4_ iron-sulfur clusters, plus various lipids and water molecules. In total, 96 chlorins were resolved, more than those resolved in the two structures of MBIC11017 PSI [73 (*9*) and 79 (*28*)]. The additionally resolved Chls d in NIES-2412 PSI are shown in Fig. 4A, and a comparison with the Chls d found in both MBIC11017 structures can be found in table S3. NIES-2412 PSI contains the same number of chlorins found in *Thermosynechococcus elongatus* BP-1 (*T. elongatus*) PSI (*29*), although Chl J03 (ligated by H39 at the C terminus of PsaJ) and M01 (found at the monomer-monomer interface near PsaM) are absent in NIES-2412 PSI. Instead, NIES-2412 PSI binds an additional Chl d (B42) in PsaB via D310 and another Chl d (I01) at the monomer-monomer interface next to PsaI (fig. S8 and discussion S1).

Fourteen of the additionally resolved Chls within NIES-2412 PSI are coordinated by PsaB (Fig. 4A). The structure of NIES-2412 PSI revealed a single transmembrane helix subunit interacting with the peripheral loops of PsaB between the fourth and fifth helices, and the seventh and eighth helices (Fig. 4B), which is not present in either MBIC11017 PSI structure. To obtain an improved ESP for this subunit, we performed symmetry expansion of the PSI trimer followed by local refinement, resulting in a map at a resolution of 2.44 Å. Although the subunit can be identified and built, the local resolution in this area is still relatively poor, possibly because of partial loss during the purification process (fig. S9). This subunit binds in a similar position to PsaX in the *T. elongatus* PSI structure (Fig. 4B). However, given its low sequence identity (~20%) and altered binding orientation, we refer to it as PsaX2 throughout the manuscript.

### Candidate red forms in NIES-2412 PSI

**Fig. 5.**
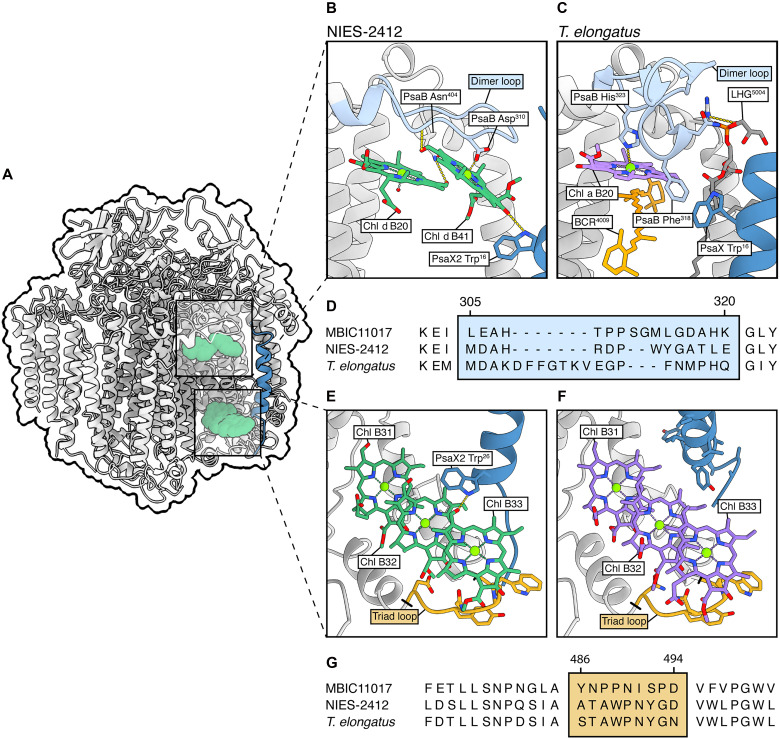
The location of the proposed additional red forms in PSI from *A. marina* NIES-2412. (**A**) View of the NIES-2412 PSI monomer parallel to the thylakoid membrane showing the location of the two proposed red forms on the cytosolic and luminal sides of PsaB (shaded in green), and the single transmembrane helix, PsaX2, that interacts with them both, shaded in blue. (**B**) The B20-B41 Chl d dimer bound by PsaB in NIES-2412. The B41 Chl d is ligated by PsaB Asp^310^ (pale blue loop) and receives a H-bond from PsaX2 Trp^16^ (to its C13^1^ carbonyl oxygen). The B20 Chl d is ligated by a water molecule and is H-bonded by PsaB Asn^404^ (to its C3 formyl group). (**C**) The same region within *T. elongatus*, showing the absence of a Chl a dimer. (**D**) Sequence alignment of PsaB in this region. (**E**) The B31-B32-B33 Chl d trimer bound by PsaB in NIES-2412. The B33 Chl d is ligated by a water molecule, which is H-bonded by PsaB Asn^489^ (orange loop). The C3 formyl group of the B33 Chl d is H-bonded by PsaX2 W26. (**F**) The B31-B32-B33 Chl a trimer in *T. elongatus*, also showing the PsaB loop and PsaX. (**G**) Sequence alignment of PsaB in this region.

Given that red forms typically originate from electronic coupling between two or more Chls (*14*, *25*, *30*), we searched for novel Chl d dimers or trimers in the NIES-2412 PSI structure. Two strong candidates emerged (Fig. 5A), both associated with unique sequence changes in PsaB and interacting with PsaX2. The first is the B31-B32-B33 trimer, bound by the loop between the seventh and eighth helices (Fig. 5E). Chl B31 is coordinated by PsaB His^465^, whereas Chls B32 and B33 are coordinated by water molecules, the latter of which is H-bonded by PsaB Asn^489^. In addition, PsaX2 Trp^26^ also provides a H-bond to the C3 formyl group of Chl B33. The center-to-center distance between these Chls is small (~8.4 Å), and their *Q*_Y_ transition dipole moments are almost parallel, favoring excitonic interactions and red-shifted absorption.

The second candidate is the B20-B41 dimer, bound by the loop between the fourth and fifth helices of PsaB (Fig. 5B and fig. S10). The B41 site is absent in *T. elongatus* PSI because of a different loop sequence and conformation that occupies the same space (Fig. 5C). Chl d B41 is ligated by PsaB Asp^310^, and its C13^1^ carbonyl oxygen is H-bonded by PsaX2 Trp^16^. Its partner, Chl d B20, is coordinated differently from other cyanobacterial PSI structures, with a water ligand replacing the histidine normally present. It is H-bonded by Asn^404^ to its C3 formyl group. In the B20-B41 dimer, Chls d are also positioned close to one another, with an interpigment separation of ~8.5 Å and an almost parallel orientation of their *Q*_Y_ transition dipole moments.

To assess the degree of exciton delocalization within both clusters, we calculated their electronic coupling values and diagonalized exciton Hamiltonians, assuming equal site energies (tables S4 to S7). The lowest-energy exciton state of the trimer and dimer are 151 and 122 cm^−1^ below the site energies of individual Chls, with their dipole strengths almost entirely distributed to these levels (98 and 93%, respectively).

Sequence alignment of PsaB from NIES-2412 and MBIC11017 reveals key differences in the B20-B41 (Fig. 5D) and the B31-B32-B33 (Fig. 5G) binding regions. MBIC11017 PsaB lacks the B41 coordinating residue, Asp^310^, and the B20 Chl is likely coordinated by His^317^, instead of a water molecule, on the basis of a histidine residue in this position in other cyanobacterial PSI structures (*29*, *31*). MBIC11017 PsaB also lacks the residue that H-bonds the water molecule that coordinates the B33 Chl d. These differences explain the absence of equivalent clusters in MBIC11017, and we therefore assign the B31-B32-B33 and the B20-B41 Chls as red forms in NIES-2412.

However, their combined oscillator strength (~5) falls short of the ~7 Chls d indicated by Gaussian deconvolution of the 77-K spectra (fig. S3). A possible candidate is the A12-A14 dimer, previously suggested as a red form in Chl a–containing PSI (*32*, *33*). In NIES-2412 PSI, these Chls d are at a center-center distance of 8.4 Å and coordinated by PsaA His216 and a water molecule H-bonded to PsaA His241. While both residues are also present in MBIC11017 PsaA, several surrounding residues found in NIES-2412 are absent in MBIC11017 (fig. S11). These residues likely stabilize the dimer, enhancing electronic coupling. Consistently, the A12-A14 dimer is not resolved in the MBIC11017 structure of Hamaguchi *et al.* (*9*) [although it is present in the structure of Xu *et al.* (*28*)].

Additional red forms candidates have been suggested in cyanobacterial PSI, including the B37-B38 and the B07-A32 dimers (*29*, *34*–*36*). Both dimers are present in NIES-2412 and MBIC11017 with identical ligands. However, in NIES-2412, nearby amino acid substitution in PsaA, PsaB, and PsaL may alter their electronic interactions and contribute to red-shifted states (fig. S12).

### Diversity of *Acaryochloris* red forms

Ulrich *et al*. (*23*) reported that NIES-2412 belongs to a clade of *A. marina* strains characterized by long-wavelength (LW) fluorescence emission in vivo (~745 nm). They also reported two other emission phenotypes: short (~723 nm) and intermediate (~730 nm) wavelengths (SW and IW, respectively). They performed phylogenomic analysis, revealing that these strains cluster according to these emission phenotypes. To understand whether the red forms identified in NIES-2412 PSI are specific to the LW group or are widespread across all strains, we performed phylogenetic analysis of PsaB and PsaX2.

**Fig. 6.**
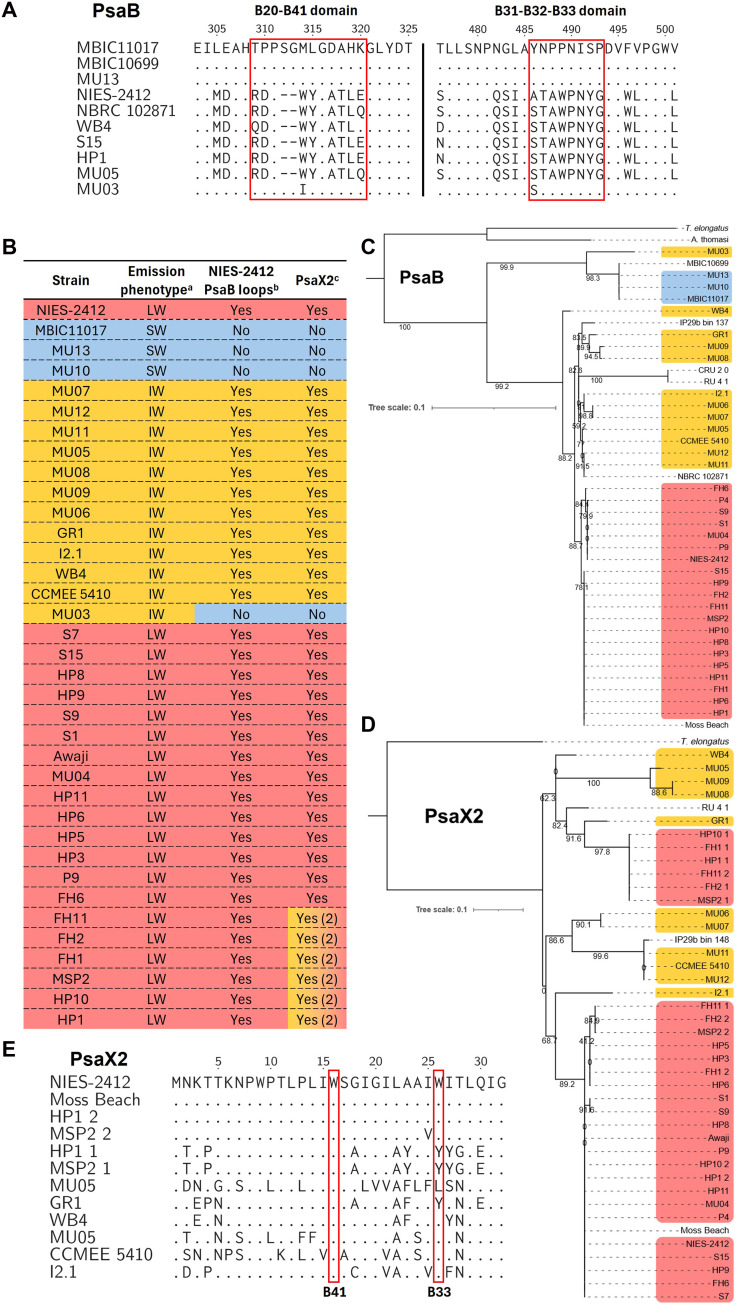
The diversity of *Acaryochloris* PsaB and PsaX2. (**A**) B20-B41 and B31-B32-B33 binding region alignment of various *Acaryochloris* strains. Red boxes highlight the loop regions that bind the B20-B41 and B31-B32-B33 Chls d. Dots indicate identical residues to the first sequence in the alignment. (**B**) Classification of *Acaryochloris* strains reported by Miller *et al.* (*37*). ^a^Strains with known emission phenotypes: short wavelength (SW): peak maximum at ~723 nm, intermediate wavelength (IW): peak maximum at ~730 nm, and LW: peak maximum at ~745 nm. ^b^PsaB sequences that contain the B20-B41 and B31-32-33 binding domains present in NIES-2412. ^c^Presence of a PsaX2 sequence within the strain genome. Strains are shaded according to their emission phenotype: SW in blue, IW in yellow, and LW in red. MU03 is an IW strain whose PsaB does not contain NIES-2412 like B20-B41 and B31-B32-B33 binding regions and does not have a PsaX2 protein, and hence, these cells are shaded in blue. Gradient yellow to red shading indicates the possession of two PsaX2 copies. (**C**) Maximum likelihood phylogeny of PsaB protein sequences. Strains are shaded according to (B). PsaB from *T. elongatus* and *Acaryochloris thomasi* were used as an outgroup. The scale represents number of substitutions per site, and branch supports are SH-like approximate likelihood ratio tests. (**D**) Maximum likelihood phylogeny of PsaX2 nucleotide sequences. Strains are shaded according to (B). PsaX from *T. elongatus* was used as an outgroup. (**E**) PsaX2 alignment of various *Acaryochloris* strains. Red boxes highlight Trp^16^ and Trp^26^, which provide H-bonds to B41 and B33 in NIES-2412 PSI. Dots indicate identical residues to the first sequence in the alignment. HP1 1 and 2 and MSP2 1 and 2 refer to the two distinct copies of PsaX2 found within these organisms.

Sequences were retrieved from the 37 strains of *A. marina* reported by Miller *et al.* (*37*) and supplemented with additional sequences from the genomes and metagenome-assembled genomes (MAGs) of *Acaryochloris* strains in National Center for Biotechnology Information (NCBI) databases. Alignment of PsaB sequences shows that the NIES-2412 B20-B41 and B31-B32-B33 binding domains are conserved in all IW and LW strains (except for the IW strain MU03) but are absent in the SW strains (Fig. 6, A and B, and fig. S13). Conserved B20-B41 and B31-B32-B33 binding loops were also found within PsaB sequences from NBRC 102871 and several *Acaryochloris* MAGs but were absent in PsaB from *A. marina* MBIC10699, for which a fluorescence phenotype is unknown. Furthermore, PsaX2 was found in all IW and LW strains (except for MU03) but not within SW strains (Fig. 6B). Six LW strains have two copies of PsaX2.

To understand the evolutionary relationships in these proteins, we constructed phylogenies from PsaB protein sequences and PsaX2 nucleotide sequences (Fig. 6, C and D). The PsaB phylogeny shows that the *Acaryochloris* strains cluster according to their emission phenotype, with the SW and MU03 PsaB sequences basal to the IW sequences, and the LW sequences emerging from the IW clade. A similar case is observed in the PsaX2 phylogeny, although no SW clade is present because of the lack of this protein in SW strains. The PsaX2 phylogeny also shows a group of PsaX2 found in LW strains that branches within the IW cluster. This group consists of PsaX2 sequences from LW strains that have two copies of PsaX2, possibly indicating that the second copy was obtained horizontally from an IW strain.

Alignment of PsaX2 protein sequences reveals that while LW versions are almost identical to NIES-2412 PsaX2, IW variants demonstrate large variation (Fig. 6E and fig. S14). All copies of PsaX2 have the B41 H-bonding residue W16, but some IW variants do not have the B33 H-bonding residue W26, instead having either a tyrosine or leucine at this position. This indicates that H-bonding to the B33 Chl d formyl group may be modified or absent in some IW strains.

## DISCUSSION

### PSI in NIES-2412 is 20 nm more red shifted than in MBIC11017

*A. marina* is the only cyanobacterial species that solely uses Chl d to absorb FRL. In the most studied strain, MBIC11017, PSI contains very few red forms, and its ability to absorb FRL is mainly due to the intrinsically red-shifted nature of Chl d. In contrast, NIES-2412 PSI extends its absorption ~20 nm further into the far red, up to ~760 nm, enabling it to capture substantially more photons between 700 and 800 nm.

Low-temperature absorption shows that this extension arises from three additional bands in the 740- to 760-nm region, originating from ~7 Chls. Pigment extraction of NIES-2412 and MBIC11017 PSI revealed no additional intrinsically red-shifted Chls [such as Chl f (*38*)]. Therefore, the extra red absorption must originate from pigment-pigment and pigment-protein interactions unique to NIES-2412.

The presence of chlorophylls absorbing at lower energy than the RC (red forms) is a hallmark of most Chl a–containing PSI complexes and allows them to use far-red photons to some extent. In plant and algal PSI, the major red forms are typically located within antenna proteins, whereas in cyanobacteria, they are integrated into the core. Some cyanobacteria also use PSI antenna proteins, such as IsiA (*39*, *40*) or Pcb proteins (*41*). The structure of NIES-2412 PSI reveals a trimeric structure that lacks any additional antenna proteins, like many cyanobacteria (*9*, *28*, *29*, *31*, *32*). Therefore, the red-shifted Chls d are located within the NIES-2412 PSI core.

### Impact of Chl d red forms on excitation energy dynamics in NIES-2412 PSI

Two pools of Chl d red forms were identified: one emitting at ~745 nm (Red1) and one at ~765 nm (Red2) with different spectral characteristics, indicating different origins. The spectrum of Red2 is broad, a feature observed in the 300-ps component from the 77-K TRF data, and the 750-nm band from the Gaussian deconvolution, indicative of extensive CT character, as is generally the case for red forms (*14*, *15*, *42*). Conversely, the spectrum of Red1 is relatively narrow, suggesting mainly excitonic character.

At 77 K, trapping from Red1 and Red2 occurs in ~50 and ~300 ps, respectively, suggesting that they are not functionally connected. This implies that the two red pools are not in direct contact and RT energy transfer between them occurs via Chls with higher energy. At RT, trapping occurs in ~16 and ~60 ps, resulting in an average trapping time of ~45 ps. The broader 60-ps decay-associated spectrum (DAS) is assigned to Red2, while the narrower 16 ps corresponds to Red 1, indicating that trapping from these red forms occurs before equilibration is complete.

Trapping from the bulk Chls d occurs in ~20 ps, similar to a wide variety of Chl a–containing PSI (*18*, *21*) and MBIC11017 PSI (*12*) The average trapping time of ~45 ps in NIES-2412 equates to a charge separation efficiency of ~98%. While the incorporation of the red forms in NIES-2412 slows down its trapping time, its charge separation efficiency remains high.

### Red form assignment in NIES-2412 PSI

To assign the red forms, we resolved the NIES-2412 PSI structure and compared it with the structures of MBIC11017 PSI. The B31-B32-B33 trimer and the B20-B41 dimer, both bound by PsaB and interacting with PsaX2, are strong candidates, as they are absent in MBIC11017 PSI (*9*, *28*). Electronic coupling calculations shows that the Chls of these clusters are strongly coupled, with oscillator strengths almost entirely distributed on the lowest-energy exciton state.

The B31-B32-B33 trimer is almost identical to a Chl a trimer previously proposed as a red site in *T. elongatus* PSI (*34*, *43*). Sequence similarity between the binding loops of NIES-2412 and *T. elongatus* strongly supports assigning this Chl d trimer to a red form. Considering that the lowest exciton state of the B31-B32-B33 trimer has an oscillator strength of ~3, and the Red2 state is formed by 1 to 2 Chls d, we assign the trimer to Red1. The B20-B41 dimer is unique to NIES-2412 as a Chl at the B41 position has not been observed in other cyanobacterial PSI structures. We thus tentatively assign it to Red2, with its strong CT character. However, unequivocal assignment of PSI red forms involving CT states requires detailed spectroscopic and theoretical analyses (*15*), which are beyond the scope of this work.

Together, these two sites account for five red-shifted Chls, leaving two additional red Chls d unassigned. Various dimers have previously been considered candidates for the red forms in Chl a–containing PSI, such as the A12-A14 (*32*, *33*), B07-A32 (*36*), and B37-B38 dimers (*44*, *45*). Chls d at these positions are found within NIES-2412 PSI and at least one of the two MBIC11017 structures, preventing us from assigning them to red forms in NIES-2412. Local sequence differences in NIES-2412 might alter the spectroscopic properties of these dimers, as seen in plant light harvesting proteins (Lhca1 versus Lhca4) where small variations red-shift emission by 40 nm (*46*).

### The role of PsaX2

The presence of a PsaX-like subunit in NIES-2412 PSI is unexpected, as it is absent in all basal cyanobacteria except for *Thermosynechococcus* spp. (*33*), a somewhat close relative of *Acaryochloris* spp. In MBIC11017, the absence of PsaX was suggested to increase structural flexibility, preventing regions of PsaB from being resolved (*9*). In contrast, these regions were resolved in NIES-2412 PSI, which may be the reason why additional Chls d were found compared to MBIC11017 (*9*, *28*), although we cannot discount that these Chls d were lost during the biochemical purification process of MBIC11017 PSI.

PsaX2 in NIES-2412 is divergent from PsaX found within Chl a PSI, but a low level of sequence identity and similarity can be noted. PsaX2 binds in a similar position as that of PsaX in *T. elongatus*, only somewhat tilted. Unlike *T. elongatus*, PsaX2 does not interact with a phospholipid. Instead, it provides H-bonds to B41 and B32 Chls d, both components of the red forms. Therefore, we suggest that PsaX2 plays a role in stabilizing the assigned red forms (via their associated PsaB loops) and in modulating their energies and electronic couplings.

### Comparison with other *A. marina* strains

Investigation into other *A. marina* strains has unveiled spectral variations compared with MBIC11017. Many strains exhibit a red-shifted shoulder in the *Q*_Y_ absorption region (*37*, *47*–*49*) and red-shifted fluorescence emission peaks (*23*, *49*, *50*). Isolated PSI complexes from strains MU05 and P4 (*23*) and NBRC 102871 (*24*) display a red-shifted shoulder to their *Q*_Y_ absorption peak and red-shifted 77-K emission peaks at ~750 nm, very similar to NIES-2412 PSI.

Comparison of PsaB from NIES-2412 with other *Acaryochloris* strains reveals that the residues responsible for coordinating and interacting with the B20-B41 and B31-B32-B33 red forms in NIES-2412 are conserved in nearly all strains, including those with known, red-shifted PSI complexes: MU05, P4, and NBRC 102871. The only exceptions are MBIC11017 and the closely related strains, MBIC10699, MU10, MU13, and MU03. Furthermore, PsaX2 is absent in these strains but is present in all *Acaryochloris* strains that have the PsaB red-form binding motifs (and for which complete genomes are available). This strong correlation suggests that most *Acaryochloris* strains have PSI complexes red shifted in a similar way to NIES-2412, with MBIC11017 and its close relatives being notable exceptions. RT in vivo emission in *Acaryochloris* strains is dominated by PSII, because of its considerably longer lifetime than PSI (*12*). We therefore find it noteworthy that both PsaB and PsaX2 genes cluster according to the emission phenotypes of Ulrich *et al.* (*23*), possibly suggesting that PSI in different *Acaryochloris* is adapted to different spectral niches within the far-red spectral region.

Last, we want to highlight the IW strain, MU03, whose PsaB lacks the B20-B41 and B31-B32-B33 binding domains and does not have a copy of PsaX2. MU03 is therefore likely to have a blue-shifted PSI complex, like MBIC11017, but a red-shifted PSII complex. Given the basal position of MU03 in the genome-wide (*23*) and PsaB phylogenies, this strain may retain an ancestral state of PSI and PSII in *Acaryochloris*.

### Outlook

In plants, broadening the absorption into the far-red region has been proposed as a potential mechanism for increasing crop yields (*51*–*53*). However, it is currently challenging to rationally design red-form binding sites into photosynthetic complexes because of various knowledge gaps. Here, we show that NIES-2412 PSI absorbs ~20 nm further into the red than MBIC11017, with only small differences that allow additional Chl d binding. By introducing these changes into plant PSI, red-form binding sites can be designed, resulting in an increase of the FRL absorption cross section.

## MATERIALS AND METHODS

### Cell growth

*A. marina* MBIC11017 was obtained from the Biological Resource Center, NITE (NBRC; Japan). *A. marina* NIES-2412 was obtained from the Microbial Culture Collection at the National Institute for Environmental Studies. Cells were grown photoautotrophically in IMK medium with 3.6% (w/v) artificial seawater (Aquaforest) at 25°C and at a constant irradiance of 20 μE m^−2^ s^−1^ using “warm white” light-emitting diodes.

### PSI isolation

Thylakoid membranes from MBIC11017 and NIES-2412 were isolated in a similar manner to that previously described (*12*, *54*), with some minor changes that are described below. Cells were centrifuged (6500*g*, 15 min) and washed in buffer A [50 mM MES, 1 M betaine monohydrate, 5 mM CaCl_2_, 5 mM MgCl_2_, and 10% (v/v) glycerol, pH 6.5 adjusted with NaOH]. Cells were centrifuged again and resuspended in buffer A containing EDTA-free protease inhibitor cocktail tablets (cOmplete, Roche), 0.2% (w/v) bovine serum albumin, and deoxyribonuclease I (50 μg ml^−1^). Cells were lysed by three passages through a prechilled French pressure cell at 150 MPa and then centrifuged (2000*g*, 10 min, 4°C) to remove cell debris. Thylakoid membranes were pelleted by ultracentrifugation (190,000*g*, 30 min, 4°C) and resuspended in buffer B [50 mM MES, 1 M betaine monohydrate, 20 mM CaCl_2_, 5 mM MgCl_2_ and 10% (v/v) glycerol, pH 6.5 adjusted with NaOH]. Membranes were washed once in buffer B and resuspended at a Chl d concentration of ~1 mg ml^−1^.

Thylakoids were solubilized for 45 min in buffer B containing 1% (w/v) *n*-dodecyl-β-maltoside. Insolubilized material was removed by centrifugation (17,000*g* 10 min at 4°C). Solubilized thylakoids were loaded on a sucrose density gradient made by freezing and thawing 0.6 M sucrose, 50 mM MES-NaOH at pH 6.5, 10 mM CaCl_2_, 5 mM MgCl_2_, and 0.04% (w/v) *n*-dodecyl-β-maltoside buffer and separated by ultracentrifugation at (270,000*g*, 15 hours, 4°C). The PSI trimer band was collected and underwent a second round of sucrose density gradient and ultracentrifugation as specified above (fig. S1).

### Pigment extraction and composition analysis

Pigments were extracted from isolated photosystem complexes with 80% acetone. The relative Chl a and d content was estimated by fitting the absorption spectrum of the pigment extract with the spectra of the isolated pigments in the same solvent (fig. S2, B and C) (*55*–*57*).

### Immunoblotting

To verify the purity of NIES-2412 PSI, it was loaded onto a 12% tricine SDS Page Gel (*58*), at a concentration of 0.5 μg of Chl. After electrophoresis, proteins were immunoblotted against PsaB and CP47 antibodies (Agrisera, Sweden) as previously described (*59*). Blots can be seen in fig. S1.

### Steady-state spectroscopy

Absorption spectra were acquired using a Varian Cary 4000 UV-VIS spectrophotometer. Low-temperature absorption spectra were obtained using a custom-built liquid nitrogen–based cooling system, for which the samples were supplemented to 70% (v/v) glycerol to prevent the formation of ice crystals. For measurements on whole cells, an integrating sphere module was used. Emission spectra were recorded using a HORIBA JobinYvon-Spex Fluorolog 3.22 spectrofluorometer, with sample concentrations maintained at an optical density below 0.05 cm^−1^ at the *Q*_Y_ peak to avoid reabsorption. For 77-K emission measurements, samples were frozen in liquid nitrogen and measured in a 1-mm path-length Pasteur pipette. CD spectra were recorded using a Chirascan CD spectrophotometer.

### Stepanov analysis

The fluorescence spectrum of the NIES-2412 PSI complex was calculated from its absorption spectrum using the Stepanov relation (*26*)

$$F\left(\nu \right)=D\left(T\right)A\left(\nu \right)\nu^{2}e^{-\frac{\mathrm{h\nu}}{K_{B}T}}$$

in which *F*(ν) is the fluorescence spectrum, *D*(*T*) is a term that only depends on the temperature [its precise meaning is given in (*26*)], *A*(ν) is the absorption spectrum, *h* is the Planck constant, *K*_B_ is the Boltzmann constant, and *T* is the temperature.

### Gaussian deconvolution

To perform the Gaussian deconvolution of the NIES-PSI 77 K *Q*_Y_ absorption spectrum, first, the second derivative of this absorption spectrum was computed. The negative peaks of this absorption were then used to guide the peak positions of the individual Gaussians during the decomposition. Specifically, during the fitting, the peak positions of the Gaussians were allowed to deviate 2 nm from the determined second derivative minima, except for the blue region of the spectrum where two Gaussians were needed to accurately fit the data.

### Streak camera measurements

The streak camera setup has been described in detail previously (*59*). Excitation pulses were provided at 400 nm at a repetition frequency of 250 kHz at an intensity of 0.8 nJ per pulse. The laser beam was focused within the sample to a spot size of ~100 μm. For the RT measurements, the sample was constantly stirred using a magnetic stirring bar. For the measurements at cryogenic temperatures, the samples were frozen in liquid nitrogen and measured in a Pasteur pipette. For the RT measurements, a streak time window of ~400 ps was chosen, yielding a time resolution of ~7 ps. For the 77-K measurement, the sample was measured using both the ~400-ps time window and a ~1.5-ns time window and analyzed simultaneously in a global analysis (vide infra).

### Global and target analysis

Global and target analyses have been performed on the TRF data using the pyglotaran Python package (*60*, *61*). In the global analysis, the time-resolved datasets ψ(λ,*t*) are fitted with a sum of exponential components that decay separately with a rate *k*, and which are convolved with the instrument response function [IRF(λ,*t*)], yielding the decay-associated spectra [DAS(λ)]

$$\psi \left(\lambda ,t\right)=\sum_{n}{\text{DAS}}_{n}\left(\lambda \right)e^{-t*k_{n}}⨂\text{IRF}\left(\lambda ,t\right)$$

The IRF was modeled as a Gaussian and its FWHM was a free fitting parameter. Charge separation efficiencies were calculated using the average trapping time from the global analysis, and the equation: ϕ_CS_ = 1 − τ_CS_/τ(2 ns), where τ_CS_ is the average trapping lifetime and τ(2 ns) is the excited-state lifetime of Chl in the absence of photochemistry (*62*).

The kinetic scheme for the target analysis is presented in Fig. 4A. To estimate the initial excitation, input vectors for the separate compartments the areas of the species-associated spectra (SAS) were constrained to be equal (*63*). In addition, the input vectors of the Red1 and Red2 compartments were forced to be minimally 2% of the Bulk input vector. Furthermore, to retrieve kinetics of the pure spectral species, the SAS of the Red1 and Red2 compartments were forced to zero for λ < 720 nm.

From the target analysis results and the total number of Chls per PSI, one can estimate the total number of Chls that are represented by the Bulk, Red1, and Red2 compartments. The ratio of forward/backward rate from the bulk to one of the Red compartments is connected to the Gibbs free energy difference of the two compartments through the Boltzmann equation

$$\frac{k_{\text{Bulk}\to \text{Red}x}}{k_{\text{Red}x\to \text{Bulk}}}=e^{-\frac{\Delta G}{K_{B}T}}$$

in which the Gibbs free energy is the sum of an enthalpic and entropic term: Δ*G* = Δ*H* − *T*Δ*S*. The enthalpic energy difference between the compartments is then extracted as the difference in energy that corresponds to the wavelength maxima of the associated SAS, which allows to determine the entropic energy difference between the compartments. The number *N* in one of the Red compartments is then computed as $N=N_{\text{Bulk}}\times e^{\frac{\mathrm{\Delta S}}{K_{B}}}$.

### Electronic coupling and excitonic dipole distribution calculation

Electronic couplings were calculated using the TrEsp method (*64*), in combination with the empirical screening expression that was derived from (Chl a–containing) PSI trimers by Eder and Renger (*65*) and the transition ESP charges for Chl d as derived by Kimura *et al.* (*66*). To retrieve the exciton levels and connected dipole strength for the triad Chls, we first diagonalized the exciton Hamiltonian, assuming equal site energies for each Chl. This yields the exciton energy levels in the form of the eigenvalues and the wave function coefficients *C_Jm_* of the individual sites *m* to each particular exciton *J* as the eigenvectors. The excitonic transition dipole moments μ*_J_* can then be calculated as

$${\vec{\mu }}_{J}=\sum_{m}C_{\mathrm{Jm}}{\vec{\mu }}_{m}$$

The transition dipole moments of the individual Chls μ*_m_* are extracted from the structure as the line connecting the NB and ND nitrogen of each Chl.

### Genome sequencing

To retrieve amino acid sequences for building the cryo-EM model, the genome of NIES-2412 was sequenced using short-read next-generation sequencing technologies. Genomic DNA from NIES-2412 was extracted using the Quick-DNA Fungal/Bacterial Miniprep Kit (Zymo Research, Irvine, CA, USA). Whole-genome sequencing was performed using the Illumina NovaSeq X plus platform and 150-bp pair-end sequencing (Novogene, Cambridge, UK). A total 3.0 GB of data was generated, of which 99.39% were effective reads. Raw reads were assembled into a draft genome using Unicycler v0.5.1 (with Spades v4.0.0) and using default parameters (*67*). The total length of the assembly was 8.13 Mbp with a theoretical sequencing coverage over 300×. The number of contigs ≥200 bp was 1148, with the longest segment being 273,609 bp and N50 72,230. Genome completeness and contamination was estimated with CheckM v1.2.4 (*68*). Overall, the NIES-2412 genome was found to be 8.05 Mbp with a completeness of 99.0% and 1.7% contamination. Annotation of the genome was carried out using Prokka v1.14.5 using default parameters (*69*) on the KBase webserver (*70*).

### Grid preparation

UltrAuFoil 2-μm hole size and 1-μm hole spacing with a 300 mesh grid were glow discharged for 100 s at 25 mA. At a temperature of 4°C and 100% humidity and in the presence of only a dim green light, 3.5 μl of PSI sample [Chl concentration (~1 mg/ml)] were applied, and grids were blotted and immediately plunge frozen in liquid ethane with a Vitrobot Mark IV (Thermo Fisher Scientific).

### Data acquisition and processing

Micrographs were acquired using a Krios I (Thermo Fisher Scientific) operated at 300 kV and a magnification of ×81,000. Images were recorded on a K3 (Gatan Inc.) with a pixel size of 1.058 Å and a dose of 40 electrons/Å^2^. Images were collected in super-resolution mode with a SelectrisX energy filter with a slit width of 20 eV. The targeted defocus range was varied from −0.8 to −2 μm using the EPU software (Thermo Fisher Scientific). A total of 12,085 movies were collected from a single grid. The frames were aligned, dose was weighted, and the contrast transfer function (CTF) was estimated in CryoSPARC v4.5.1 (*71*). Micrographs were curated by removing those with CTF fits worse than 10 Å and other unsuitable parameters. The subset obtained 77% of the initial micrographs and was used to handpick ~500 PSI-type particles across the defocus range. Particles used to train topaz (*72*) templates were picked across the entire dataset, yielding 334,934 picks. After multiple rounds of topaz training, two-dimensional (2D) classification, and ab initio refinement, one major class emerged, one corresponding to PSI trimers. Duplicated particles were removed from the stack, and ~20,000 particles was used for homogeneous refinement to obtain an initial map at a resolution of ~3 Å and to confirm C3 symmetry. After multiple rounds of per-particle CTF refinement and local motion correction, a set complete set of 151,833 particles was used to perform nonuniform refinement imposing C3 symmetry, producing a map at a global resolution of 2.63 Å based on GS-FSC at a cutoff of 0.143 [Protein Data Bank (PDB) ID: 9SK3]. The Guinier’s analysis reports an estimated B-factor of 68.4. To obtain further definition of the PsaX2 subunit, local refinement was performed on symmetry-expanded particles. A mask encompassing the other two subunits was used to subtract the signal from the other two monomers. The reconstruction of the PSI monomer has a GS-FSC of 2.44 Å (PDB ID: 9S6P).

### Model building

The trimeric PSI from MBIC11017 (PDB ID: 7COY) (*9*) was fitted to the ESP map using the Phenix software suite (*73*), mutated to the correct sequence with Chainsaw (Phenix), and refined in Coot (*74*). Each subunit density was evaluated amino acid by amino acid, and the model was then refined in real space in Phenix.

### Sequence alignments

Contigs from the *A. marina* strains reported in (*37*) were retrieved from Logan (*75*). Nucleotide sequences for PsaB and PsaX2 were searched for using blastn (*76*) with NIES-2412 query sequences and then translated. Additional sequences were found within the NCBI sequence databases. With the exception of PsaI and PsaX2, protein sequences in the nonredundant (nr) database were searched for using blastp. In the case of PsaI, sequences were found via tblastn in both the nucleotide (nt) and whole-genome shotgun (WGS) databases and subsequently translated. In the case of PsaX, sequences were found using a nucleotide input via blastn in both the nt and WGS databases and subsequently translated. Sequences were aligned using the Muscle algorithm (*77*) within SeaView (*78*) using the default settings. Alignments were visualized using TEXshade (*79*).

## Supplementary Materials

### This PDF file includes:

Figs. S1 to S19 Tables S1 to S7 Supplementary Discussion S1
