# Supplementary material for: Far-red chlorophyll d clusters extend photosystem I absorption toward the red limit
Source: Sci Adv. 2026 Jun 10;12(24):eaed7355. doi: 10.1126/sciadv.aed7355 (PMC13251853; doi:10.1126/sciadv.aed7355)
Supplement: Supplementary file 1 — Figs. S1 to S19 Tables S1 to S7 Supplementary Discussion S1 [file sciadv.aed7355_sm.pdf]

Science Advances
AAAS

Supplementary Materials for

Far-red chlorophyll d clusters extend photosystem I absorption toward the red limit

Thomas J. Oliver *et al.*

Corresponding author: Roberta Croce, r.croce@vu.nl

Sci. Adv. **12**, eaed7355 (2026)
DOI: 10.1126/sciadv.aed7355

This PDF file includes:

Figs. S1 to S19
Tables S1 to S7
Supplementary Discussion S1

Supplementary Figures

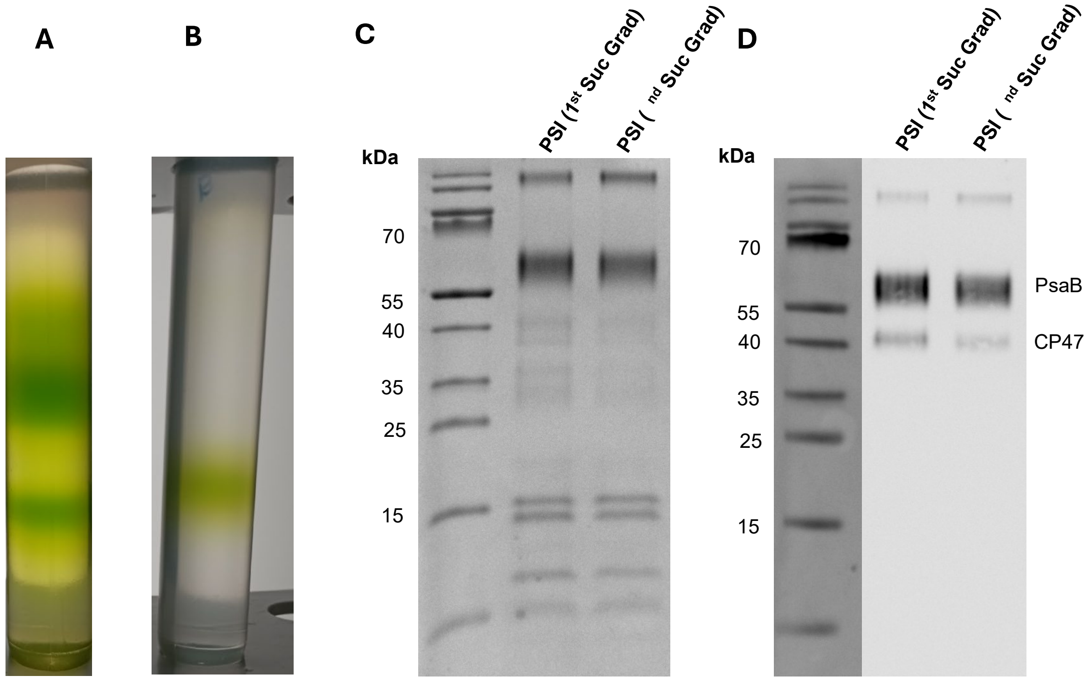

Figure S1. Purification of *Acaryochloris marina* NIES-2412 Photosystem I. (A) Sucrose density gradient of NIES-2412 solubilised thylakoids. The lowest major band contained NIES-2412 PSI. **(B)** Sucrose density gradient of cleaned NIES-2412 PSI. **(C)** 12% tricine SDS PAGE gel of NIES-2412 PSI (Lane 1), and cleaned NIES-2412 PSI. Samples were loaded at a Chl concentration of 0.5 µg **(D)** Immunoblot using PsaB and CP47 antibodies to show minimal contamination of PSII in the NIES-2412 PSI. Samples were loaded at a Chl concentration of 0.5 µg.

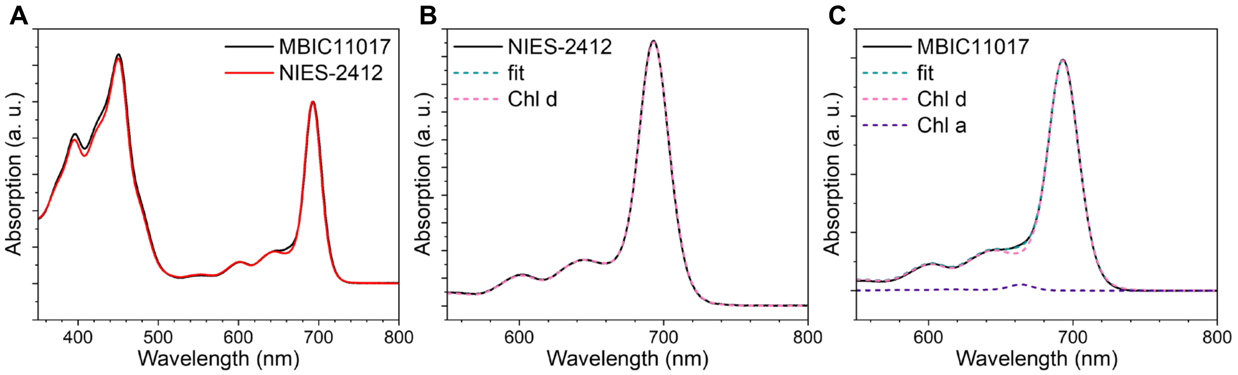

Figure S2. Absorption spectrum of pigments extracted from Acaryochloris marina NIES-2412 and MBIC11017 PSI.

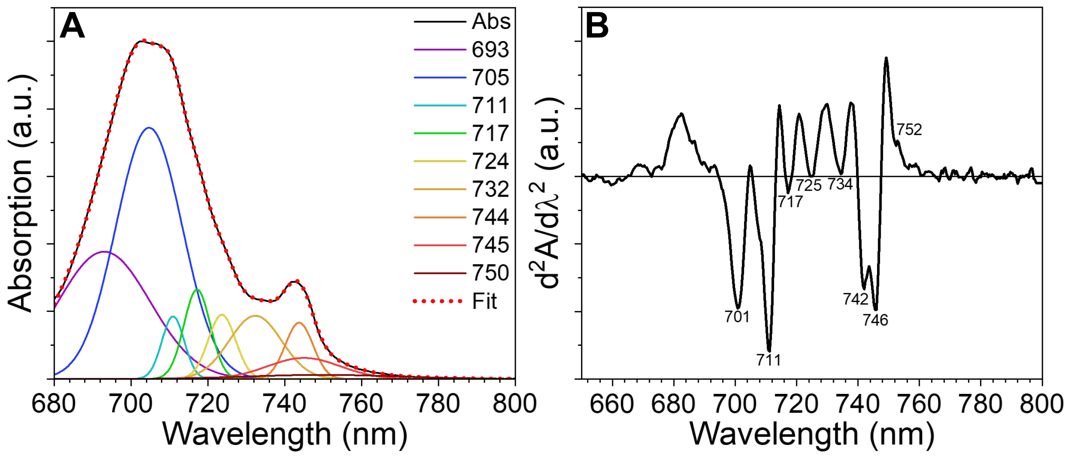

Figure S3. Gaussian deconvolution and Second-derivative of the NIES-2412 PSI 77 K absorption spectrum. (A) The Gaussian deconvolution of the NIES-2412 PSI 77 K absorption spectrum was guided by the negative peaks of second derivative spectrum **(B),** in particular: the peak positions of the Gaussians were allowed to deviate two nanometers from the determined second derivative minima, except for the blue region of the spectrum where two Gaussians were needed to accurately fit the data.

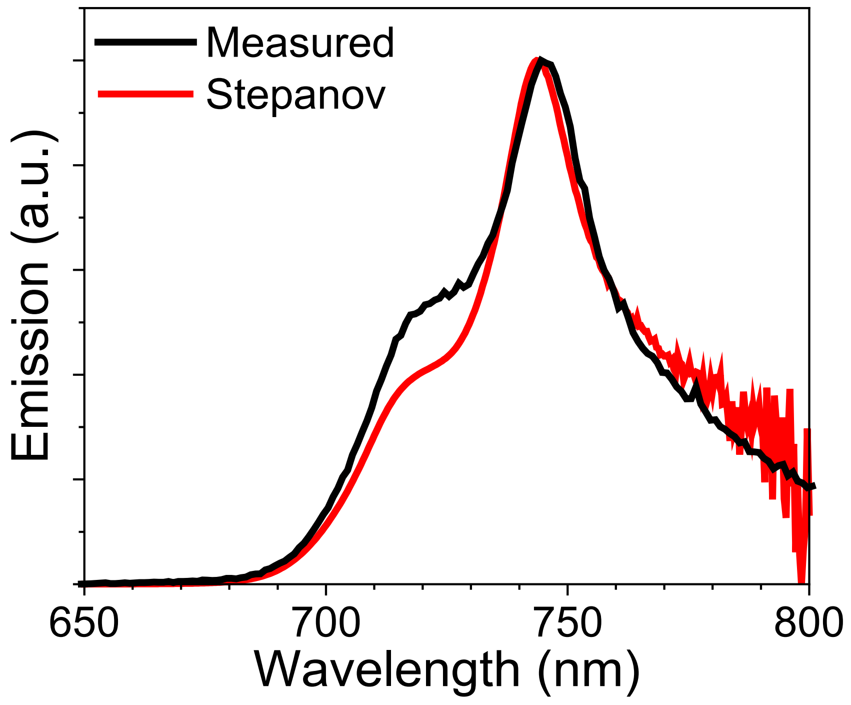

Figure S4. Measured – and Stepanov predicted RT emission spectrum for the NIES-2412 PSI complex.

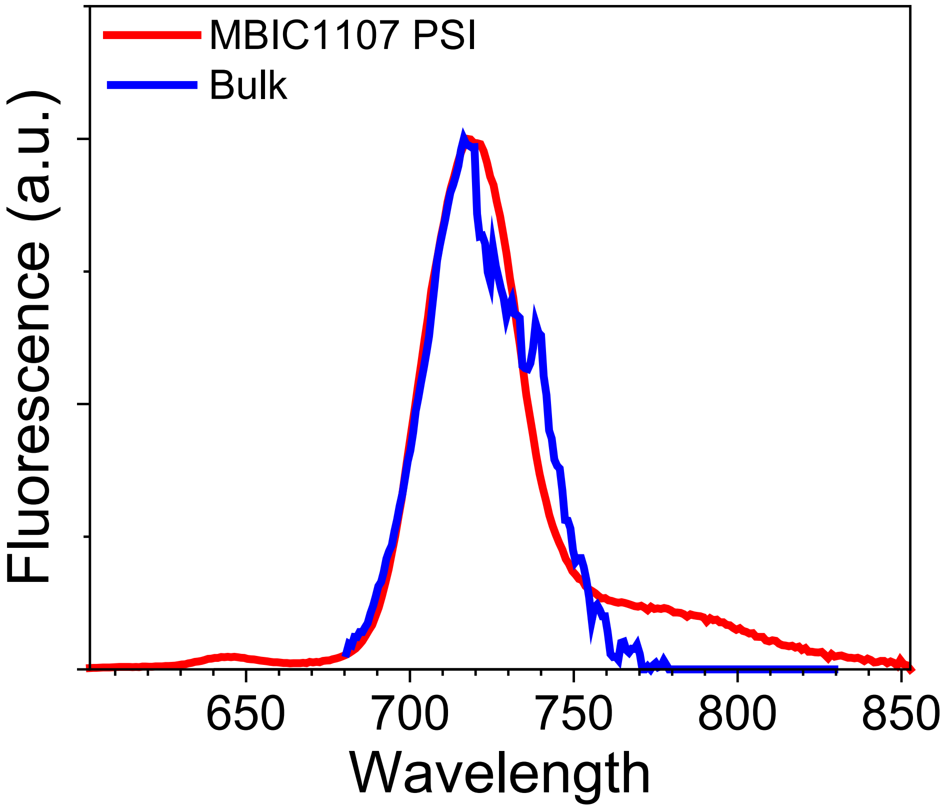

Figure S5. Comparison of the Bulk compartment SAS from the NIES-2412 PSI target analysis and the emission spectrum of MBIC1107 PSI (*12*).

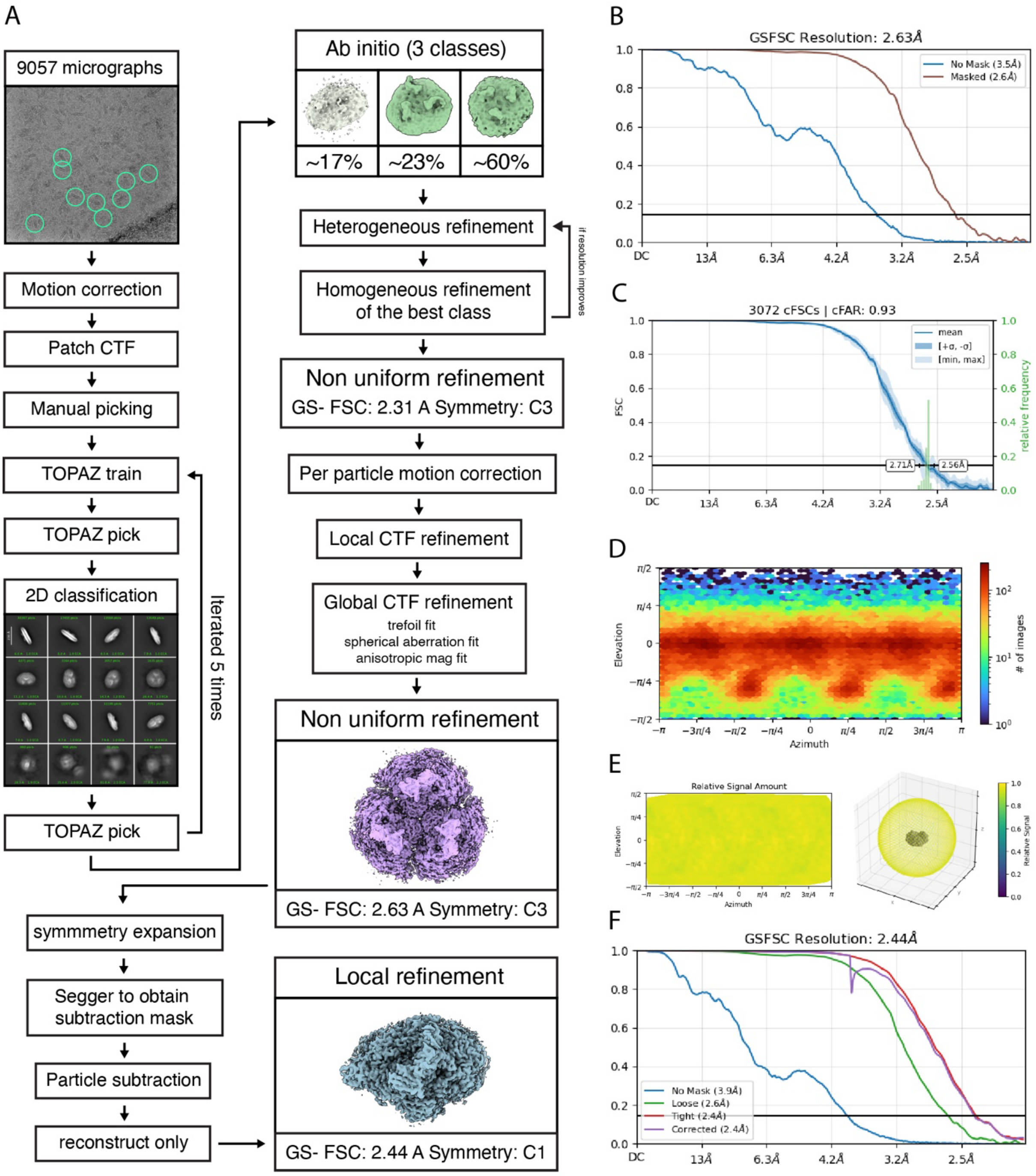

Figure S6. Processing workflow and reconstruction diagnostic plots for trimeric and monomeric PSI. **(A)** Processing workflow to obtain the final reconstruction of the trimeric PSI. **(B)** GS-FSC curve for the trimeric PSI. (pdb_00009SK3) **(C)** cFSC curves for the trimeric PSI complex. **(D)** Viewing direction distribution of the particle stack. **(E)** relative signal amount for each of the viewing directions. **(F)** GS-FSC curve for the locally refined monomer of NIES-2412 PSI (pdb_00009S6P).

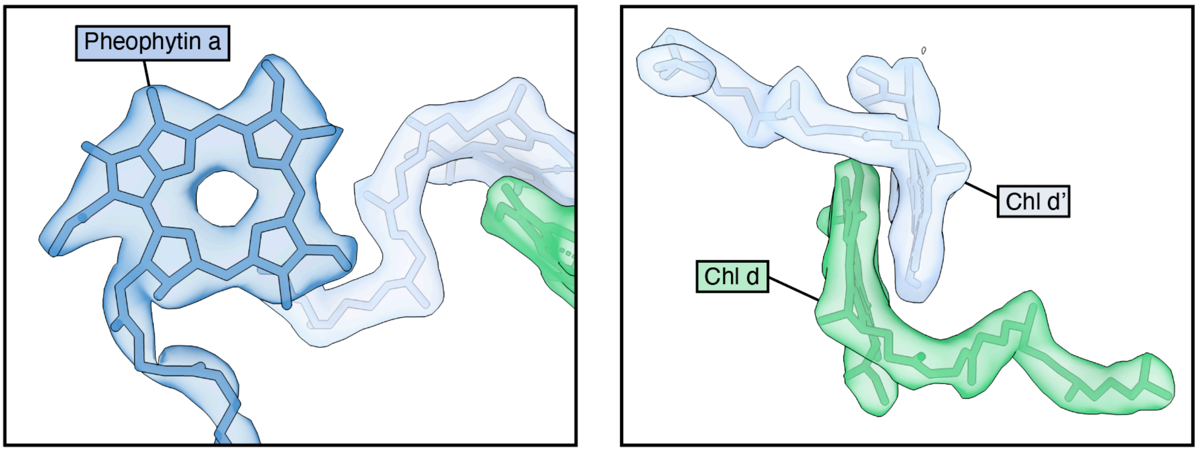

Figure S7. The presence of pheophytin *a* and Chl d/d'in the Photosystem I (PSI) reaction centre from *Acaryochloris marina* NIES-2412. The two pheophytin *a* molecules occupying A_{0A} and A_{0B} can be identified by the absence of density corresponding to the Mg^{2+} ion centrally ligated in chlorophylls. Chlorophyll *d'* is the C13 epimer of chlorophyll *d*, resulting in inversion of the orientation of the bulky substituent relative to the chlorin ring plane, which is structurally distinguishable.

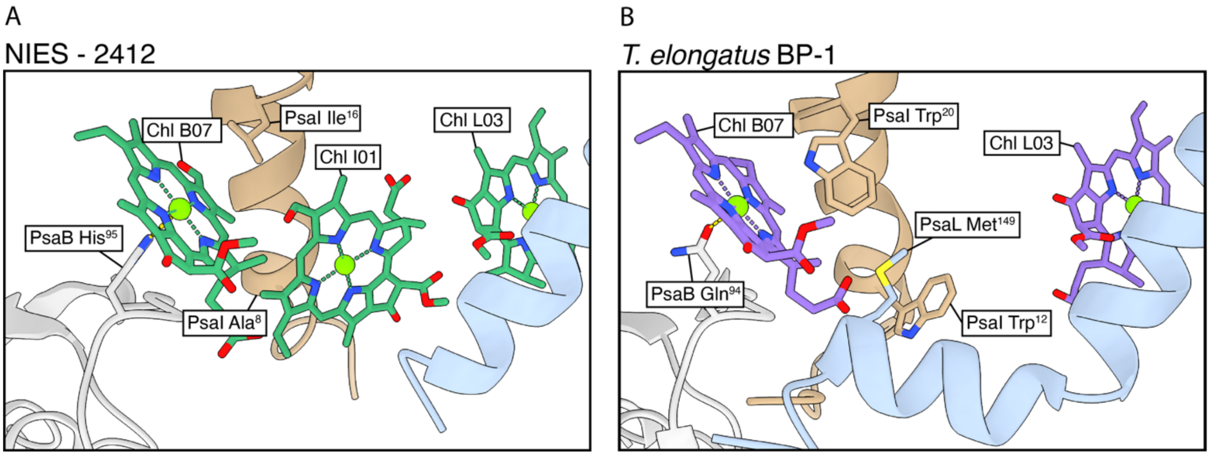

Figure S8. The location of the additional Chl *d* bound by PsaI Photosystem I (PSI) from *Acaryochloris marina* NIES-2412. (A) Chl *d* I01 is found between the N-terminus of PsaI and the C-terminus of PsaL in *A. marina* NIES-2412 PSI. **(B)** The equivalent location in (PSI) from *T. elongatus*, demonstrating the lack of a Chl at this position.

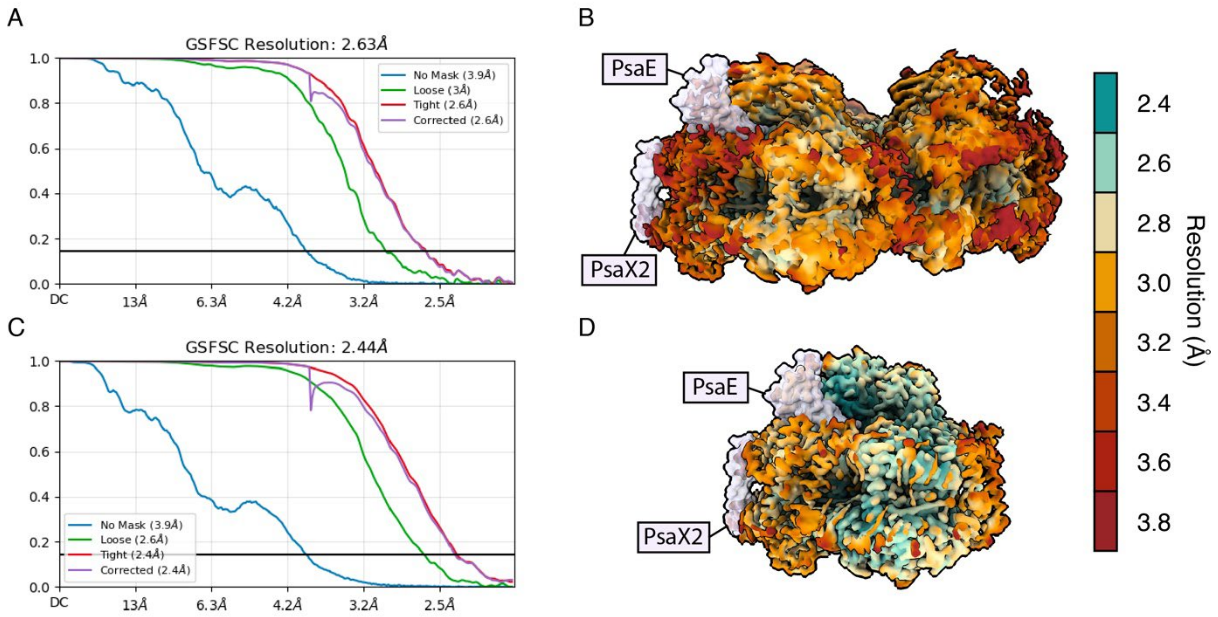

Figure S9. Local resolution of the trimeric and local refined structure of Chl d PSI from NIES-2412. (A) GS-FSC curve for the trimer of NIES-2412 PSI (PDB ID: 9SK3) **(B)** Mapping of the local resolution of 9SK3 according to the colorbar on the right, the PsaX2 and PsaE subunits, that present poor density, are highlighted in pink. **(C)** GS-FSC curve for the locally refined monomer of NIES-2412 PSI (PDB ID: 9S6P) **(D)** Mapping of the local resolution of 96SP according to the colorbar on the right, the PsaX2 and PsaE subunits, that present poor density, are highlighted in pink. The lower local resolution does not influence the conclusion of this work.

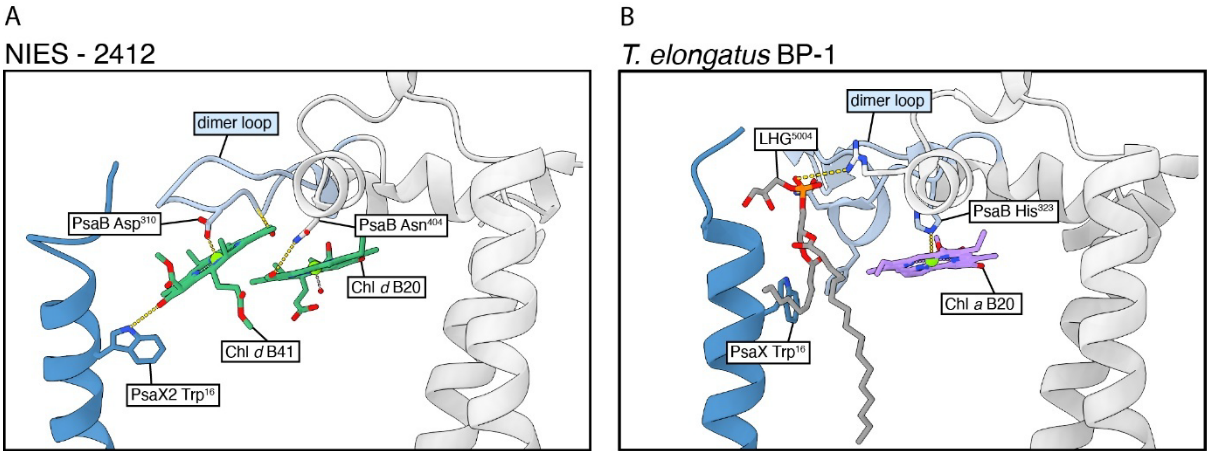

Figure S10. Alternative angle of the B20-B41 Chl *d* dimer in NIES-2412 PSI (A) and the corresponding perspective in *T. elongatus* PSI (B).

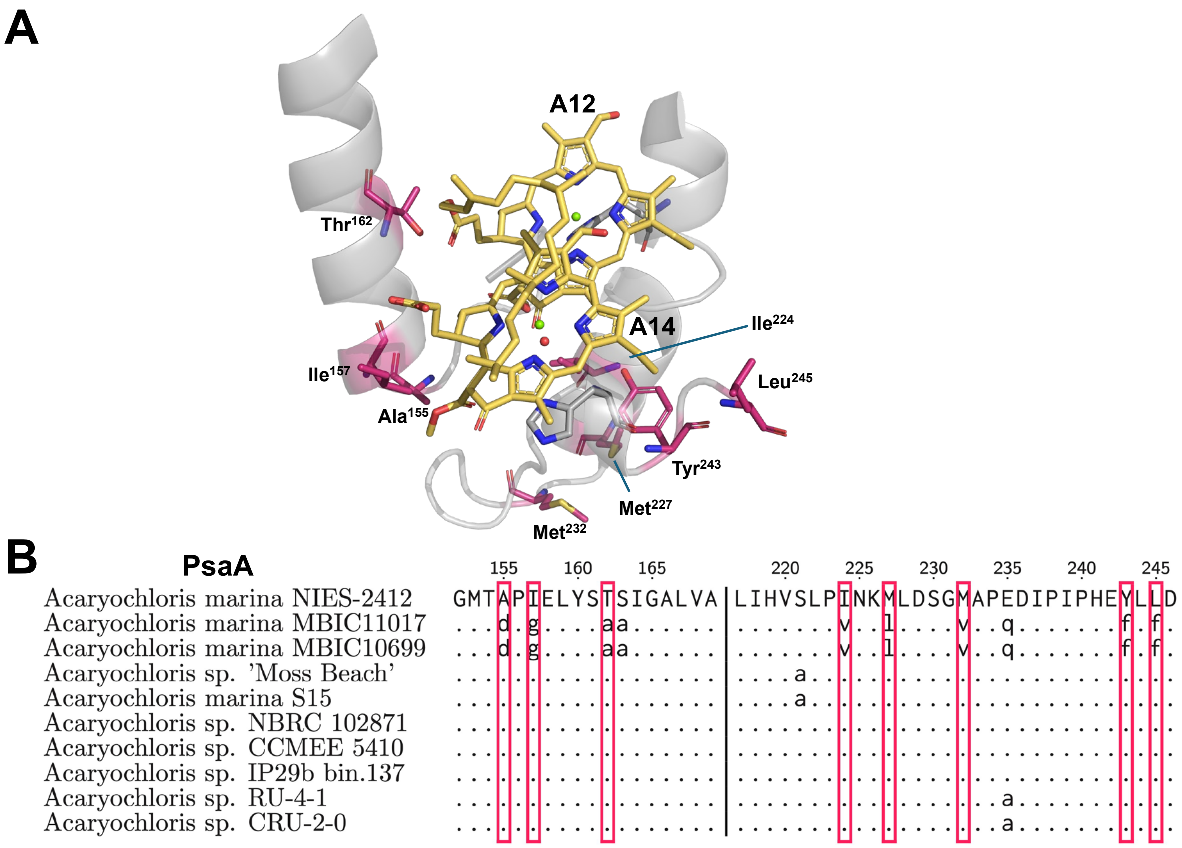

Figure S11. PsaA differences between NIES-2412 and MBIC11017 PSI around the A12-A14 Chl *d* dimer. (A) The A12-A14 Chl *d* dimer in NIES-2412 PSI. PsaA residues highlighted in pink are different from those found in MBIC11017 PsaA. **(B)** Sequence alignment of PsaA from NIES-2412, MBIC11017 and various Acaryochloris strains. Red boxes highlight the PsaA residues that are different between NIES-2412 and MBIC11017 in the vicinity of the A12-A14 Chl *d* dimer. Dots indicate identical residues to the first sequence in the alignment.

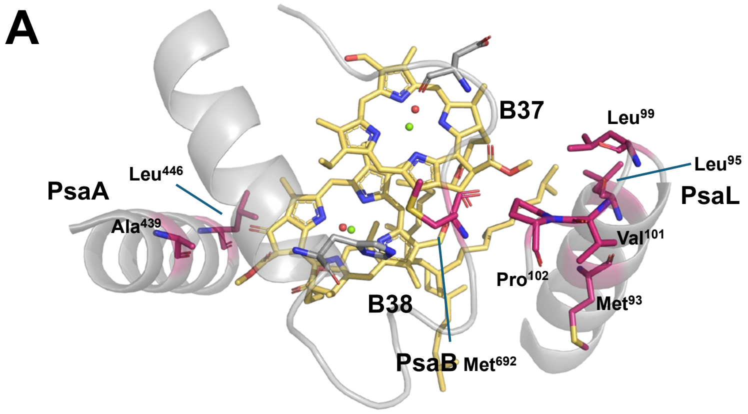

B **PsaA**

Strain	430–450
Acaryochloris marina NIES-2412	MLRHRDTIIAHLNWVCLFLGF
Acaryochloris marina MBIC11017	.........s......i....
Acaryochloris marina MBIC10699	.........s......i....
Acaryochloris sp. 'Moss Beach'	....
Acaryochloris marina S15	....
Acaryochloris sp. NBRC 102871	....
Acaryochloris sp. CCMEE 5410	....
Acaryochloris sp. IP29b bin.137	....
Acaryochloris sp. RU-4-1	.........s...........
Acaryochloris sp. CRU-2-0	.........s...........

C **PsaB**

Strain	685–705
Acaryochloris marina NIES-2412	HQRTPLANLVMWKDKPVALSIVQGRL
Acaryochloris marina MBIC11017	..........T...............
Acaryochloris marina MBIC10699	..........T...............
Acaryochloris sp. 'Moss Beach'	....
Acaryochloris marina S15	....
Acaryochloris sp. NBRC 102871	....
Acaryochloris sp. CCMEE 5410	....
Acaryochloris sp. IP29b bin.137	....
Acaryochloris sp. RU-4-1	....
Acaryochloris sp. CRU-2-0	....

D **PsaL**

Strain	90–105
Acaryochloris marina NIES-2412	IVLTVAMQLYSSLSVPKPRPS
Acaryochloris marina MBIC11017	...S..I.I...V.SA..L..
Acaryochloris marina MBIC10699	...S..I.I...V.SA..L..
Acaryochloris sp. 'Moss Beach'	....
Acaryochloris marina S15	....
Acaryochloris sp. NBRC 102871	...S..I.I...V.SA..L..
Acaryochloris sp. CCMEE 5410	...S..I.I...V.SA..H..
Acaryochloris sp. IP29b bin.137	...S..I.I...V.SA..L..
Acaryochloris sp. IP29b bin.148	...S..I.I...V..A.....
Acaryochloris sp. SU-5-25	VI....IS..I.V.....IAT
Acaryochloris sp. RU-4-1	VI....IS..I.V.....IAT
Acaryochloris sp. CRU-2-0	VI....IS..I.V.....IAT

Figure S12. Differences in PsaA, PsaB, and PsaL between NIES-2412 and MBIC11017 PSI around the B37-B38 Chl *d* dimer. (A) The B37-B38 Chl *d* dimer in NIES-2412 PSI. Residues highlighted in pink are different from those found in MBIC11017 PSI. Sequence alignments of PsaA **(B)**, PsaB **(C)**, and PsaL **(D)** from NIES-2412, MBIC11017 and various Acaryochloris strains are shown below. Red boxes highlight

the residues that are different between NIES-2412 and MBIC11017 in the vicinity of the B37-B38 Chl *d* dimer. Dots indicate identical residues to the first sequence in the alignment.

```
PsaB          B20-B41 domain              B31-B32-B33 domain

                  305  310  315  320  325     480  485  490  495  500
MBIC11017     EILEAH[TPPSGMLGDAHK]GLYDT | TLLSNPNGLA[YNPPNISP]DVFVPGWV
MBIC10699     ......[............]..... | ..........[........]........
NIES-2412     ..MD..[RD.--WY.ATLE]..... | S.....QSI.[ATAWPNYG]..WL...L
Moss˙Beach    ..MD..[RD.--WY.ATLE]..... | N.....QSI.[STAWPNYG]..WL...L
S15           ..MD..[RD.--WY.ATLE]..... | N.....QSI.[STAWPNYG]..WL...L
NBRC˙102871   ..MD..[RD.--WY.ATLQ]..... | S.....QSI.[STAWPNYG]..WL...L
CCMEE˙5410    ..MD..[RD.--WY.ATLQ]..... | S.....QSI.[STAWPNYG]..WL...L
IP29b˙bin˙137 ..MD..[RD.--WY.ATLQ]..... | K.....QSI.[STAWPNYG]..WL...L
RU˙4˙1        ..MD..[KD.--WY.ATLQ]..... | G...D..SI.[STAWPNYG]..WL...L
CRU˙2˙0       ..MD..[KD.--WY.ATLQ]..... | G...D..SI.[STAWPNYG]..WL...L
MU07          ..MD..[RD.--WY.ATLQ]..... | S.....QSI.[STAWPNYG]..WL...L
MU13          ......[............]..... | ..........[........]........
MU10          ......[............]..... | ..........[........]........
MU12          ..MD..[RD.--WY.ATLQ]..... | S.....QSI.[STAWPNYG]..WL...L
MU11          ..MD..[RD.--WY.ATLQ]..... | S.....QSI.[STAWPNYG]..WL...L
MU05          ..MD..[RD.--WY.ATLQ]..... | S.....QSI.[STAWPNYG]..WL...L
MU08          ..MDV.[RD.--WY.ATLQ]..... | N.....QSI.[STAWPNYG]..WL...L
HP8           ..MD..[RD.--WY.ATLE]..... | N.....QSI.[STAWPNYG]..WL...L
MSP2          ..MD..[RD.--WY.ATLE]..... | N.....QSI.[STAWPNYG]..WL...L
MU09          ..MDV.[RD.--WY.ATLQ]..... | N.....QSI.[STAWPNYG]..WL...L
HP10          ..MD..[RD.--WY.ATLE]..... | N.....QSI.[STAWPNYG]..WL...L
HP1           ..MD..[RD.--WY.ATLE]..... | N.....QSI.[STAWPNYG]..WL...L
HP9           ..MD..[RD.--WY.ATLE]..... | N.....QSI.[STAWPNYG]..WL...L
S9            ..MD..[RD.--WY.ATLE]..... | S.....QSI.[ATAWPNYG]..WL...L
S1            ..MD..[RD.--WY.ATLE]..... | S.....QSI.[ATAWPNYG]..WL...L
MU100         ..MD..[RD.--WY.ATLE]..... | S.....QSI.[ATAWPNYG]..WL...L
MU04          ..MD..[RD.--WY.ATLE]..... | S.....QSI.[ATAWPNYG]..WL...L
MU06          ..MD..[RD.--WY.ATLQ]..... | S.....QSI.[STAWPNYG]..WL...L
GR1           ..MD..[RD.--WY.TTLQ]..... | K.....ESI.[STAWPNYG]..WL...L
MU03          ......[.....I......]..... | ..........[S.......]........
HP11          ..MD..[RD.--WY.ATLE]..... | N.....QSI.[STAWPNYG]..WL...L
HP6           ..MD..[RD.--WY.ATLE]..... | N.....QSI.[STAWPNYG]..WL...L
HP5           ..MD..[RD.--WY.ATLE]..... | N.....QSI.[STAWPNYG]..WL...L
HP3           ..MD..[RD.--WY.ATLE]..... | N.....QSI.[STAWPNYG]..WL...L
FH11          ..MD..[RD.--WY.ATLE]..... | N.....QSI.[STAWPNYG]..WL...L
FH6           ..MD..[RD.--WY.ATLE]..... | S.....QSI.[ATAWPNYG]..WL...L
FH2           ..MD..[RD.--WY.ATLE]..... | N.....QSI.[STAWPNYG]..WL...L
FH1           ..MD..[RD.--WY.ATLE]..... | N.....QSI.[STAWPNYG]..WL...L
I2.1          ..MD..[RD.--WY.ATLQ]..... | S.....QSI.[STAWPNYG]..WL...L
WB4           ..MD..[QD.--WY.ATL.]..... | D.....QSI.[STAWPNYG]..WL...L
P9            ..MD..[RD.--WY.ATLE]..... | S.....QSI.[ATAWPNYG]..WL...L
P4            ..MD..[RD.--WY.ATLE]..... | S.....QSI.[ATAWPNYG]..WL...L
```

Figure S13. Sequence alignment of PsaB from various *Acaryochloris* strains. (A) B20-B41 and B31-B32-B33 binding region alignment of MBIC11017, NIES-2412, and other *Acaryochloris* strains. Red boxes highlight the loop regions responsible for binding the additional Chls *d* i.e. B41 and B33, respectively. Dots indicate identical residues to the first sequence in the alignment.

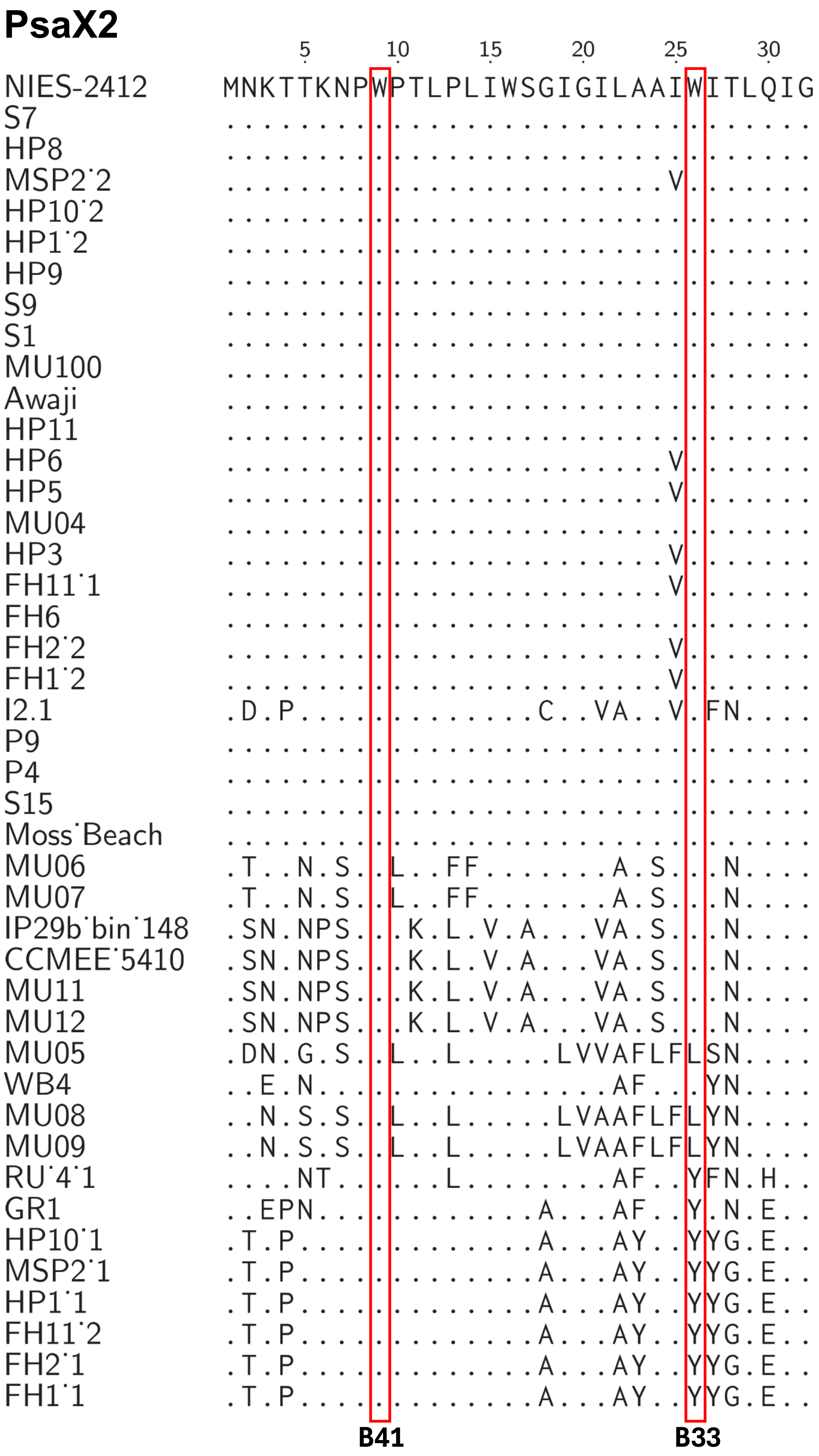

Figure S14. Sequence alignment of PsaX2 from various *Acaryochloris* strains. Red boxes highlight W16 and W26 which provide H-bonds to B41 and B33 in NIES-2412 PSI. Dots indicate identical residues to the first sequence in the alignment.

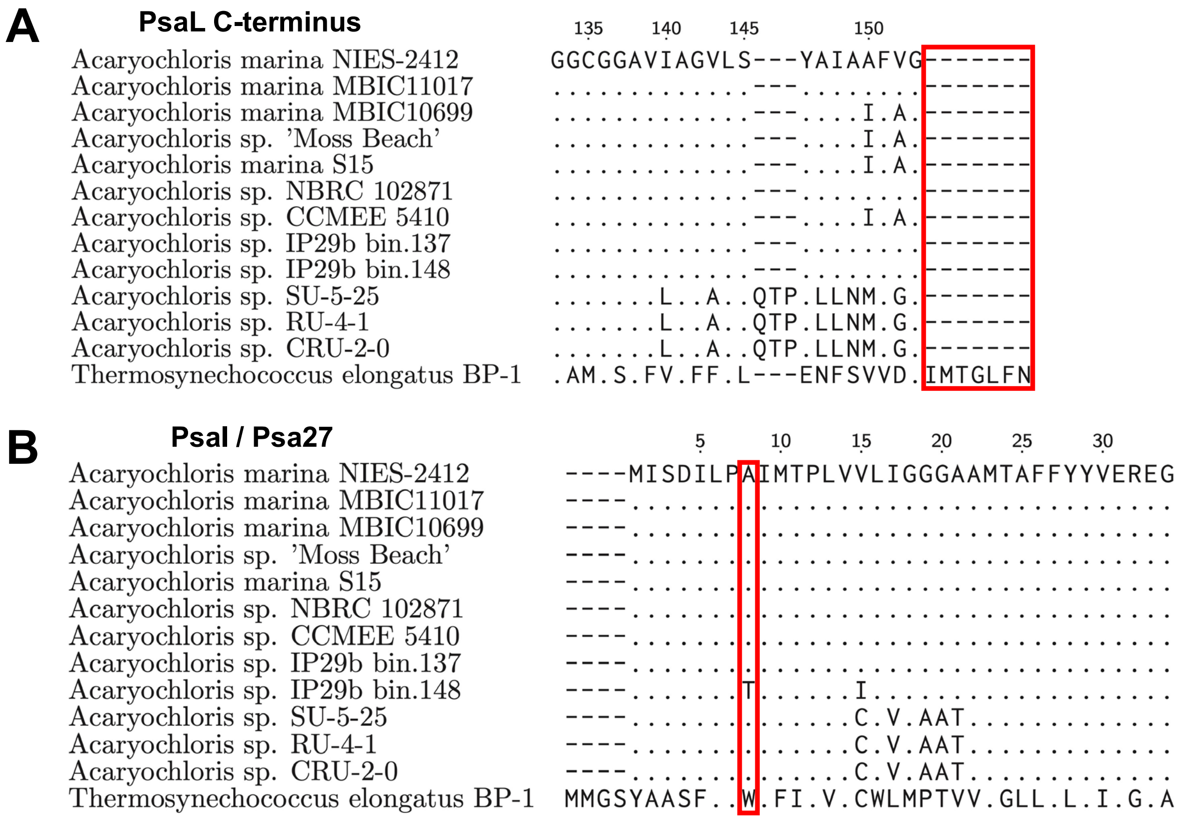

Figure S15. Sequence alignment of PsaL and PsaI (Psa27) from various *Acaryochloris* strains compared to *Thermosynechococc*us elongatus BP-1. (A) PsaL C-terminus alignment of NIES-2412, MBIC11017 and various other *Acaryochloris* strains, compared to *T. elongatus*. The red box highlights the truncated C-terminus of PsaL in *Acaryochloris* strains vs. *T. elongatus*. **(B)** PsaI (referred to as Psa27 by Hamaguchi et al (*9*) in MBIC11017) alignment of NIES-2412, MBIC11017 and various other Acaryochloris strains, compared to *T. elongatus*. The red-box highlights Ala^{8} in *Acaryochloris* strains which is found as Trp^{12} in *T. elongatus*. Dots indicate identical residues to the first sequence in the alignment.

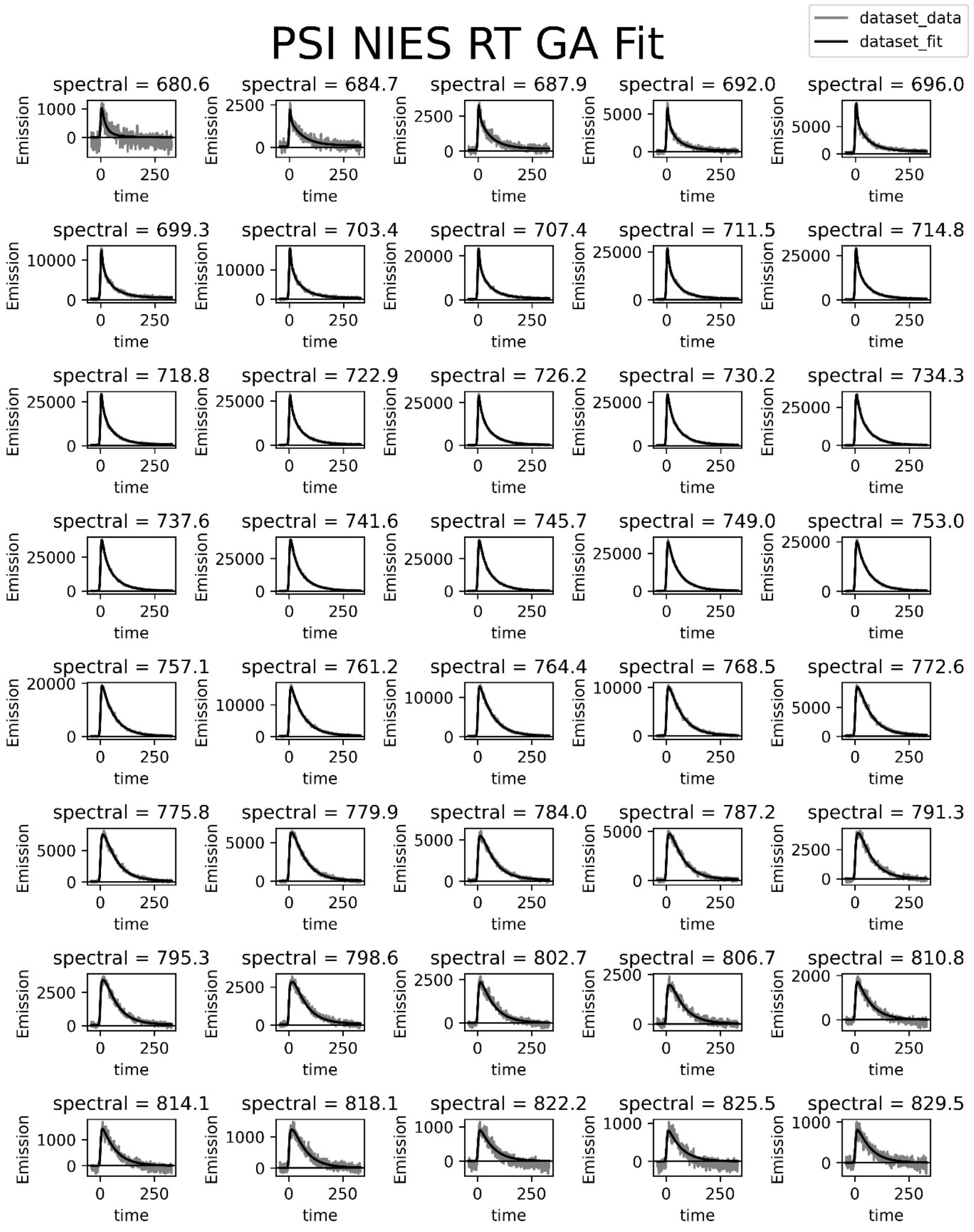

Figure S16. Fitting results for the global analysis of the RT time-resolved fluorescence experiment on NIES-2412 PSI.

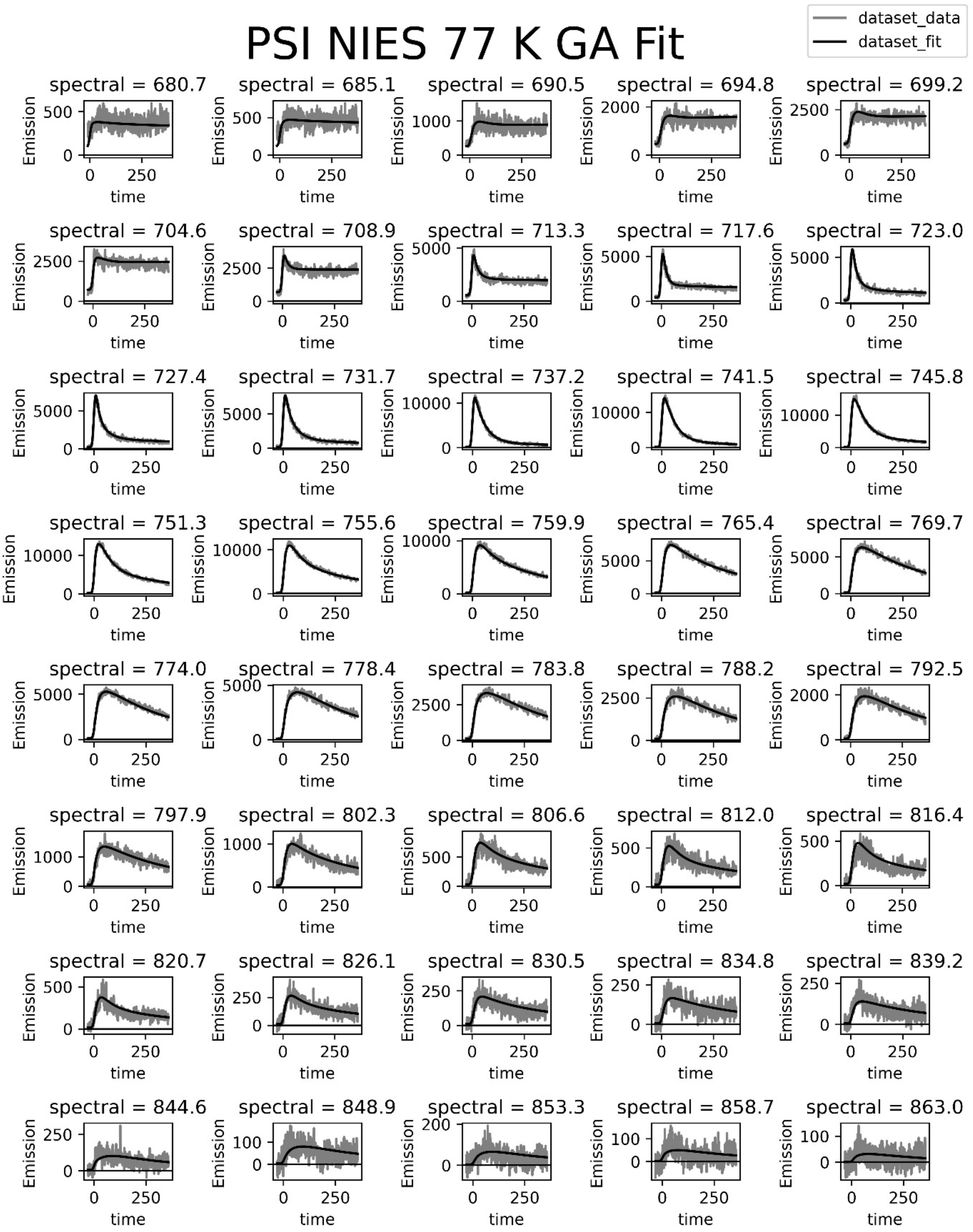

Figure S17. Fitting results for the global analysis of the 77 K time-resolved fluorescence experiment on NIES-2412 PSI for the ~400 ps time-window data.

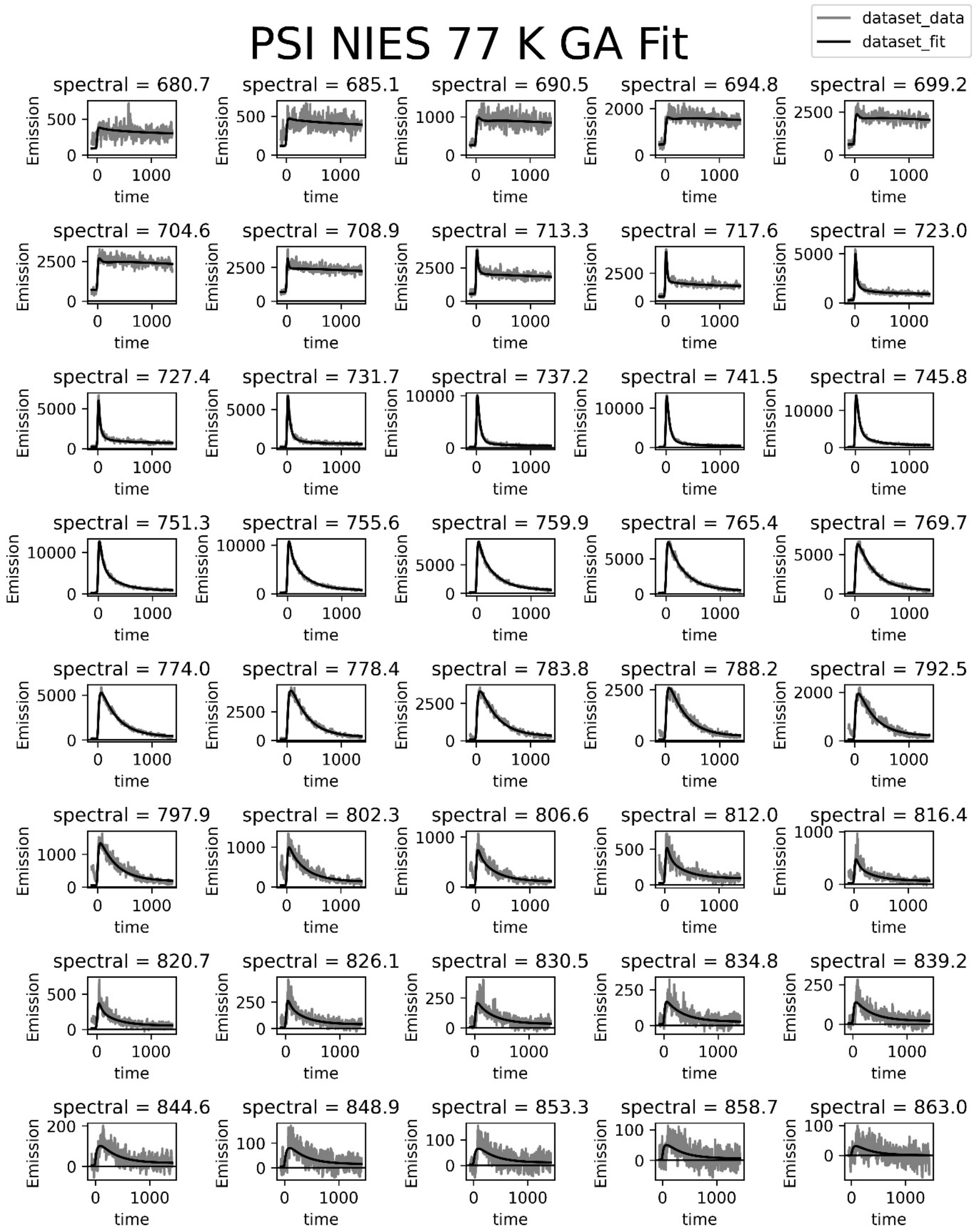

Figure S18. Fitting results for the global analysis of the 77 K time-resolved fluorescence experiment on NIES-2412 PSI for the ~1.5 ns time-window data.

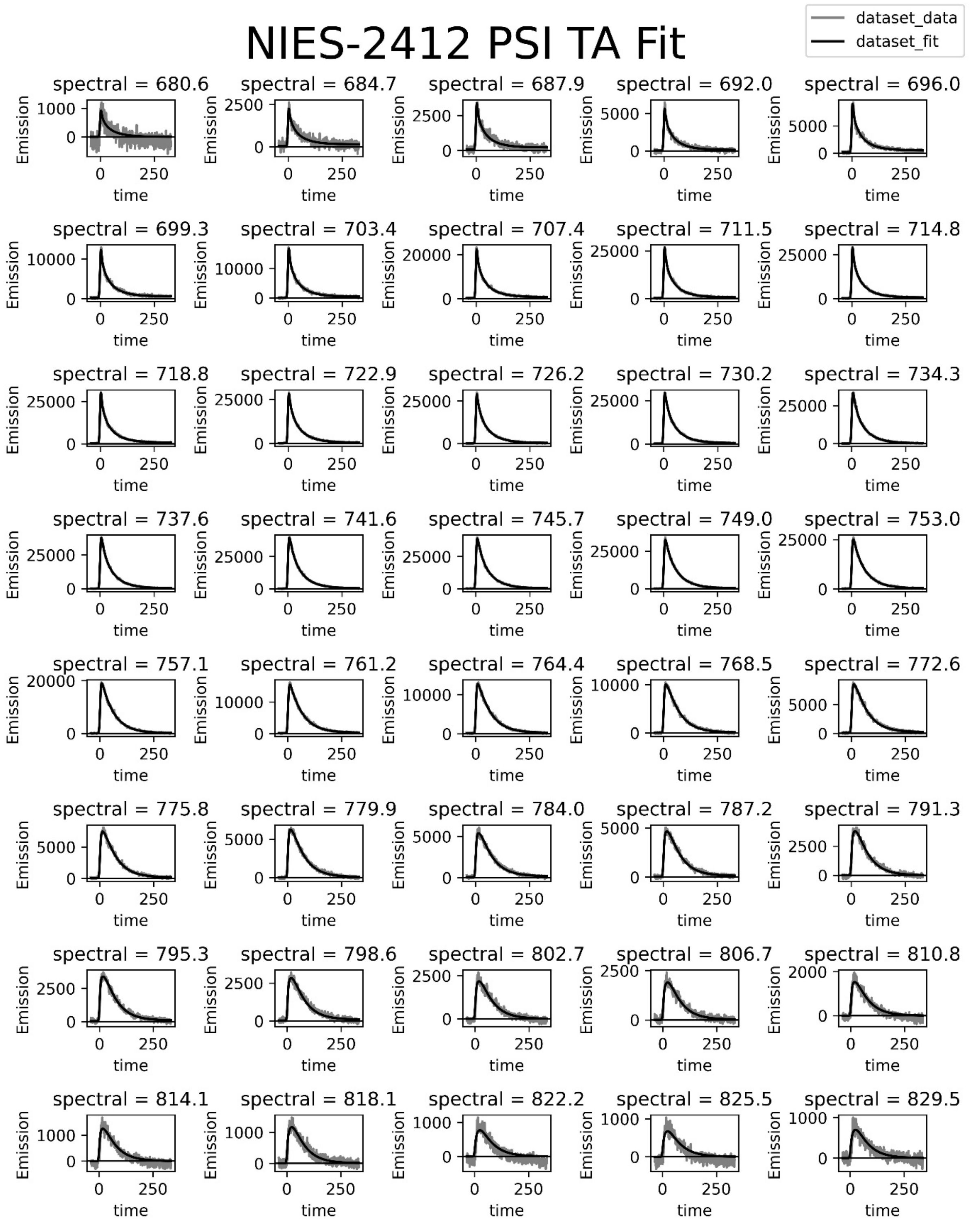

Figure S19. Fitting results for the target analysis of the RT time-resolved fluorescence experiment on NIES-2412 PSI.

Supplementary Tables

	**Bulk**	**Red1**	**Red2**
**λ_{max} (nm)**	716.4	744.9	757.1
**ΔH (k_BT)**	0	-2.62	-3.68
**ΔG (k_BT)**	0	0.25	0.74
**TΔS (k_BT)**	0	-2.87	-4.42
**N_{Chl}**	90	5	1

Table S1. Thermodynamic properties of NIES-2412 PSI arising from the target analysis. The wavelength maxima are retrieved as the maxima of the corresponding SAS of the compartments. These maxima are used to calculate the enthalpic energy difference (ΔH) between the compartments, with the Bulk as a reference. The Gibbs free energy difference (ΔG) between the compartments is calculated from the rate equilibria that arose from the target analysis, and the entropic energy difference (TΔS) follows from the equation ΔH- ΔG = TΔS. The number of Chls in the Red compartments is then calculated using $N_{Chl} = N_{Bulk}\, e^{\frac{\Delta S}{K_B}}$. We have used a temperature of 293 K for the calculations.

Data collection

Microscope	Krios I
Camera	K2
Magnification	81000x
Voltage (kV)	300
Electron exposure (e-/Å^2)	40
Defocus range (μm)	-0.8 to -2.0
Pixel size (Å)	1.058
Energy filter	Selectris (20 eV)
Exposures	12085
Image format	EER

Data processing

Box size	450 px
Initial particles (no.)	334934
Final particles (no.)	151833
Symmetry	C3
Map resolution (Å)	2.63
Map sharpening *B* factor	-68.4

Model Refinement

Refinement package	PHENIX
Initial model used	7COY
Real/reciprocal space	Real Space
Resolution cutoff	2.70

Model Validation

MolProbity score	1.29
ClashScore	5.35
Bond length R.M.S.D. (Å)	0.002

Bond angles (°)	0.555
Poor rotamers	0.24%
Favored rotamers	93.48%
Ramachandran outliers	0.05%
Ramachandran favored	98.67%

Table S2. Data collection, processing and model building and validation parameters.

	CHL D			CHL A	H-BOND TO C3		H-BOND TO C13	
CHL INDEX	9SK3	7COY	7DWQ	1JB0	NIES-2412	MBIC11017	NIES-2412	MBIC11017
**A1011**	yes	yes	yes	yes			A/Ser741	A/Ser741
**A1022**	yes	yes	yes	yes				
**A1013**	yes	yes	yes	yes			A/Tyr694	A/Tyr694
**A1101**	yes	yes	yes	yes				
**A1102**	yes	yes	yes	yes				
**A1103**	yes	yes	yes	yes	A/His57	A/His56	A/Asn356	A/Ans356
**A1104**	yes	yes	yes	yes	A/Trp360	A/Trp360		
**A1105**	yes	no	yes	yes				
**A1106**	yes	yes	yes	yes			A/Ser142	A/Ser142
**A1107**	yes	yes	yes	yes				
**A1108**	yes	no	no	yes				A/Tyr179
**A1109**	yes	yes	yes	yes			A/Lys72	A/Lys71
**A1110**	yes	yes	yes	yes			A/Trp190	A/Trp189
**A1111**	yes	no	yes	yes			A/His76	A/His77
**A1112**	yes	no	yes	yes				
**A1113**	yes	yes	yes	yes				
**A1114**	yes	no	yes	yes				
**A1115**	yes	yes	yes	yes	A/Tyr264	A/Tyr264		
**A1116**	yes	yes	yes	yes				
**A1117**	yes	yes	yes	yes	A/Tyr305	A/Tyr305		
**A1118**	yes	yes	yes	yes			A/Thr314	A/Thr314
**A1119**	yes	yes	yes	yes				
**A1120**	yes	no	yes	yes				
**A1121**	yes	yes	yes	yes				
**A1122**	yes	yes	yes	yes				
**A1123**	yes	yes	yes	yes			A/His200	A/His200
**A1124**	yes	yes	yes	yes			A/Gln488	A/Gln488
**A1125**	yes	yes	yes	yes	A/Thr507	A/Ser507		
**A1126**	yes	yes	yes	yes			A/Trp87	A/Trp87
**A1127**	yes	yes	yes	yes	A/Tyr401		A/Tyr377	A/Tyr377
**A1128**	yes	yes	yes	yes			A/Arg575	A/Arg575
**A1129**	yes	yes	yes	yes				
**A1130**	yes	yes	yes	yes	A/Trp443	A/Trp443	L/Thr19	L/Thr19
**A1131**	yes	yes	yes	yes			A/Trp443	A/Trp443
**A1132**	yes	yes	yes	yes			A/Arg467	A/Arg467
**A1133**	yes	yes	no	yes				
**A1134**	yes	yes	no	yes				
**A1135**	yes	yes	yes	yes			A/Gln488	A/Gln488
**A1136**	yes	yes	yes	yes				
**A1137**	yes	yes	yes	yes				
**A1138**	yes	yes	yes	yes	F/Ser105	F/Ser105	B/Ser421	B/Ser423
**A1139**	yes	yes	yes	yes	A/Trp150	A/Trp150	F/Ser105	F/Ser105
**A1140**	yes	yes	yes	yes			A/Gln724	A/Gln724

**A1801**	yes	yes	yes	yes				
**B1021**	yes	yes	yes	yes				
**B1012**	yes	yes	yes	yes				
**B1023**	yes	yes	yes	yes			B/Tyrr670	B/Tyrr670
**B1201**	yes	yes	yes	yes			I/Tyr29	I/Tyr29
**B1202**	yes	yes	yes	yes			B/His335	B/His337
**B1203**	yes	yes	yes	yes				
**B1204**	yes	yes	yes	yes				
**B1205**	yes	yes	yes	yes			B/Ser118	B/Ser118
**B1206**	yes	yes	yes	yes	A/Glu461	A/Asn461	B/Asp114	B/Asp114
**B1207**	yes	yes	yes	yes				
**B1208**	yes	yes	yes	yes				
**B1209**	yes	no	no	yes			B/Trp167	B/Trp167
**B1210**	yes	no	yes	yes			B/His50	B/His50
**B1211**	yes	yes	yes	yes				
**B1212**	yes	yes	yes	yes				
**B1213**	yes	yes	yes	yes				
**B1214**	yes	yes	yes	yes				
**B1215**	yes	yes	yes	yes	B/Tyr284	B/Tyr284	B/Tyr123	B/Tyr122
**B1216**	yes	no	no	yes				
**B1217**	yes	no	no	yes			B/Thr293	B/Thr293
**B1218**	yes	no	no	yes				
**B1219**	yes	no	no	yes				
**B1220**	yes	no	no	yes				
**B1221**	yes	no	no	yes				
**B1222**	yes	yes	yes	yes	B/Tyr385		B/Gln374	B/Gln374
**B1223**	yes	yes	yes	yes				
**B1224**	yes	yes	yes	yes			B/Trp60	B/Trp60
**B1225**	yes	yes	yes	yes			B/Tyr356	B/Tyr358
**B1226**	yes	yes	yes	yes				
**B1227**	yes	yes	yes	yes			B/Arg408	B/Arg410
**B1228**	yes	yes	yes	yes	B/Trp422	B/Trp424		
**B1229**	yes	yes	yes	yes			B/Trp422	B/Trp424
**B1230**	yes	yes	yes	yes	B/Asp433			
**B1231**	yes	no	no	yes				
**B1232**	yes	no	no	yes			B/Asn489	B/Asn489
**B1233**	yes	no	no	yes			X/Trp26	
**B1234**	yes	yes	yes	yes	B/Tyr370	B/Tyr372	B/Gln464	B/Gln464
**B1235**	yes	yes	yes	yes				
**B1236**	yes	yes	yes	yes				
**B1237**	yes	yes	yes	yes			B/Asn442	B7Asn442
**B1238**	yes	yes	yes	yes	B/Thr18	B/Thr18		
**B1239**	yes	yes	yes	yes				
**B1241**	yes	no	no	no			X/Trp16	
**L1501**	yes	yes	yes	yes				
**L1502**	yes	yes	yes	yes				

**L1503**	yes	yes	yes	yes			L/Tyr56	L/Tyr56
**X1701**	no	no	no	yes				
**F1701**	yes	no	no	no				
**I1601**	yes	no	yes	no				
**J1301**	yes	no	yes	yes				
**J1302**	yes	yes	yes	yes				
**J1302**	no	no	no	yes				
**K1401**	yes	yes	no	yes				
**K1402**	yes	yes	no	yes				
**M1601**	no	no	no	yes				

Table S3. Chlorophyll sites present in Chl *a* and Chl *d* containing PSI complexes. The presence (or absence) of Chl at a specific position in the PSI structures from NIES-2412 (PDB ID: 9SK3, present work), MBIC11017 (7COY (*9*) and 7DWQ (*28*)), and *T. elongatus* (1JB0 (*29*)) is indicated in either green (or orange). Additionally, H-bonding residues to either the C3 formyl group or C13 keto group are indicated.

	**B31**	**B32**	**B33**
**B31**	0	-112	-25
**B32**	-112	0	-80
**B33**	-25	-80	0

Table S4. Exciton Hamiltonian for the B31, B32 & B33 Chl triad. Electronic coupling values are listed in the off-diagonal and the relative site-energies are arranged on the diagonal. All values in cm^{-1}.

**Exciton energy level (cm^{-1})**	**Dipole strength distribution (%)**
-151	95
24	4
127	1

Table S5. Exciton levels and excitonic dipole strength distributions for the exciton Hamiltonian of Tab. S3.

	B20	B41
**B20**	0	122
**B41**	122	0

Table S6. Exciton Hamiltonian for the B20 & B41 dimer. Electronic coupling values are listed in the off-diagonal and the relative site-energies are arranged on the diagonal. All values in cm^{-1}.

Exciton energy level (cm^{-1})	Dipole strength distribution (%)
-122	93
122	7

Table S7. Exciton levels and excitonic dipole strength distributions for the exciton Hamiltonian of Tab. S5.

Supplementary Discussion

Discussion S1. An Acaryochloris specific PsaI-bound Chl *d*.

The NIES-2412 PSI structure shows the presence of an additional Chl *d* (I01), coordinated by PsaI and located between PsaB of one monomer and PsaL of the adjacent one (**Fig. S8**). There is no Chl present at this position in any resolved Chl *a*-PSI structure. Inspection of the *T. elongatus* PsaI and PsaL sequences reveals that Chl binding at this position is blocked primarily by the presence of PsaI Trp^{20}, as well as PsaL Met^{149}. In NIES-2412 PsaI, there is an alanine instead of a tryptophan at this position, and the PsaL C-terminus is truncated, allowing the binding of Chl *d* here. These changes are also present in MBIC11017 (**Fig. S15**), and indeed a Chl *d* is observed in the same position in the structure of Xu et al. (28).

Given the close proximity of this Chl *d* to the monomer-monomer interface, and the conservation of this site across different *Acaryochloris* strains, it could be argued that this Chl may play a role in inter-monomer EET in NIES-2412 PSI. To test this hypothesis, we calculated the excitation energy transfer rate between this Chl and its nearest neighbour in an adjoining monomer (i.e. Chl L03) using the framework laid out in Croce and van Amerongen (13) (taking the parameters for Chl *a*→Chl *a* EET). The calculated energy transfer rate between these Chls is very small (0.02 ps^{-1}), which would not allow for efficient EET across monomers, given that this Chl is much more strongly connected to other Chls within its own monomer (the calculated energy transfer rate from Chl I01 to Chl B07 is 2.9 ps^{-1}). Interestingly, the centre-to-centre distance between the I01 and B07 Chls *d* is only 11.7 Å. It is possible

that the presence of I01 could alter the electronic interaction between the B07-A32 Chls *d* (a potential red form).
